# Surface co-crystallization with an organic single crystal enables vapochromic and luminescent detection of phenols

**DOI:** 10.1039/d6sc05755a

**Published:** 2026-08-18

**Authors:** Ivan Bondarenko, Kaleb Hood, Elani J. Cabrera-Vega, Aminata Dioume, Alaric Shaw, Josephine Bicknell, Simon J. Teat, Nicholas S. Settineri, Andrea M. Goforth, Stephanie S. Lee, Jun Jiao, Gonzalo Campillo-Alvarado

**Affiliations:** a Department of Chemistry, Reed College Portland OR USA gcampillo@reed.edu; b Department of Mechanical and Materials Engineering, Portland State University Portland OR USA; c Department of Chemistry, Portland State University Portland OR USA; d Molecular Design Institute, Department of Chemistry, New York University New York New York 10003 USA; e Advanced Light Source, Lawrence Berkeley National Laboratory Berkeley CA 94720 USA; f Department of Chemistry, University of California, Berkeley Berkeley California 94720-1460 USA

## Abstract

We present a novel approach for the vapochromic and luminescent detection of phenols through surface co-crystallization with single crystals of 9,10-bis((*E*)-2-(pyridin-4-yl)vinyl)anthracene BP4VA. Upon exposure to 2,4,6-trifluorophenol (2,4,6-TFP), 3,4,5-trifluorophenol (3,4,5-TFP), and phenol (PhOH) vapor, BP4VA crystals exhibit significant changes in color and fluorescence intensity, offering a sensitive detection method. Detailed structural insights into intermolecular interactions are obtained from single-crystal and powder X-ray diffraction, as well as FT-IR and ^1^H NMR spectroscopies. Scanning electron microscopy and energy dispersive X-ray spectroscopy demonstrate the reversibility of these changes, with crystal morphology and surface composition nearly returning to near-pristine single crystals of BP4VA after annealing. The recoverability of single crystals enables a promising platform for the detection of volatile organic compounds.

## Abstract 2

Single crystals of an organic semiconductor undergo surface co-crystallization with phenol vapors, producing color and luminescence changes. The process is reversible, enabling solid-state sensing through hydrogen bonding at the crystal surface.

## Graphical abstract

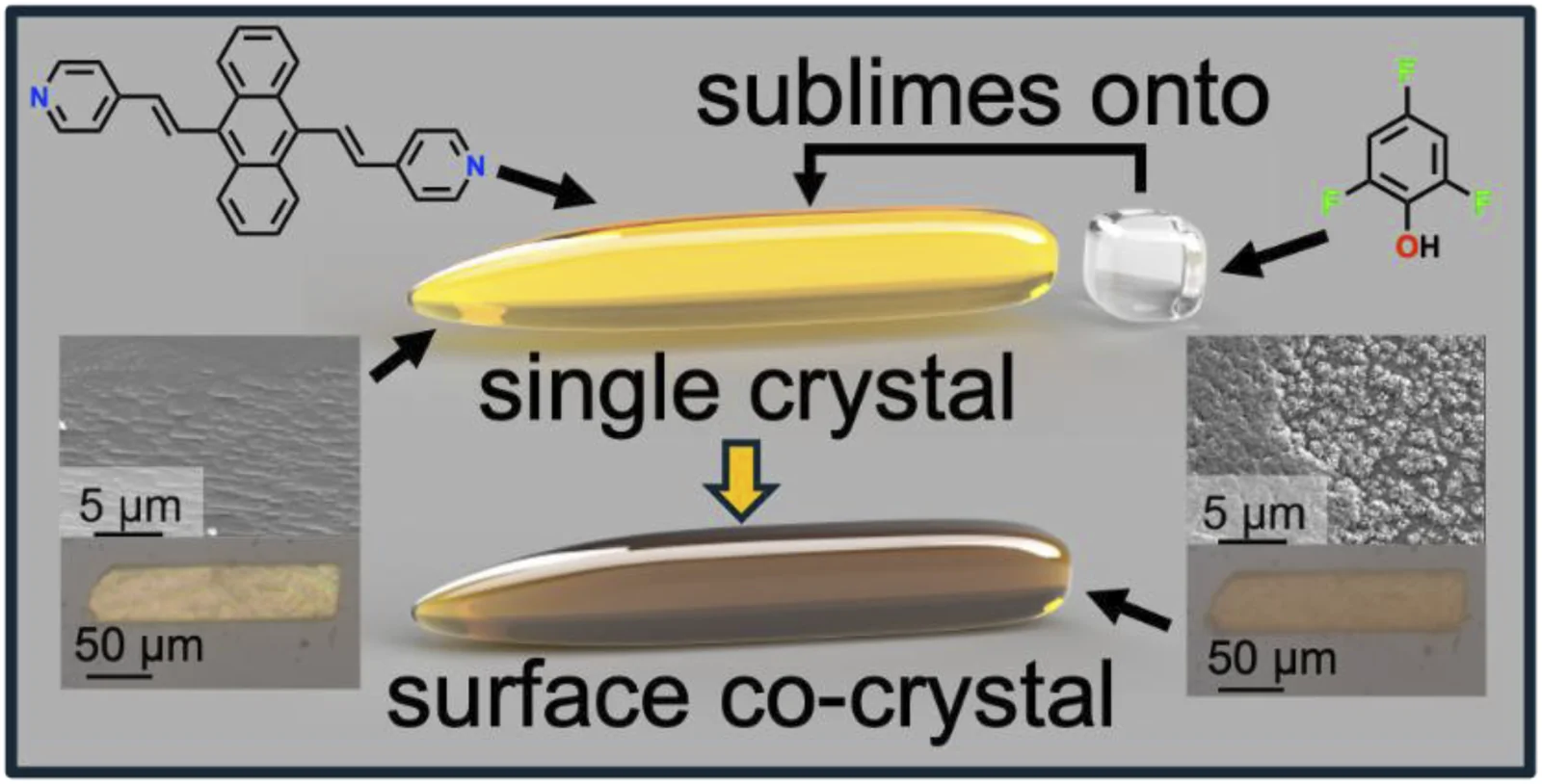

## 1 Introduction

Detection and recognition of volatile organic compounds (VOCs) have become a growing need for healthcare and environmental monitoring.^1–3^ Several industrial VOCs are harmful pollutants to human health and the environment, with a growing concern over respiratory diseases and cancer.^4^ While gas chromatography coupled with mass spectrometry (GC-MS) is the most frequently used technique for detecting VOCs, large instrument dimensions, high costs, and the need for specialized training limit its applicability.^5^ As a result, developing smaller-scale detection techniques remains a priority for fabricating portable next-generation sensing devices.^5^ Single crystals offer a unique sensing platform due to their reduced size and ordered molecular arrangement, making them desirable for VOC detection in wearable devices,^6–9^ yet their application remains underexplored compared to thin films or nanostructured materials.^6^

**Scheme 1:**
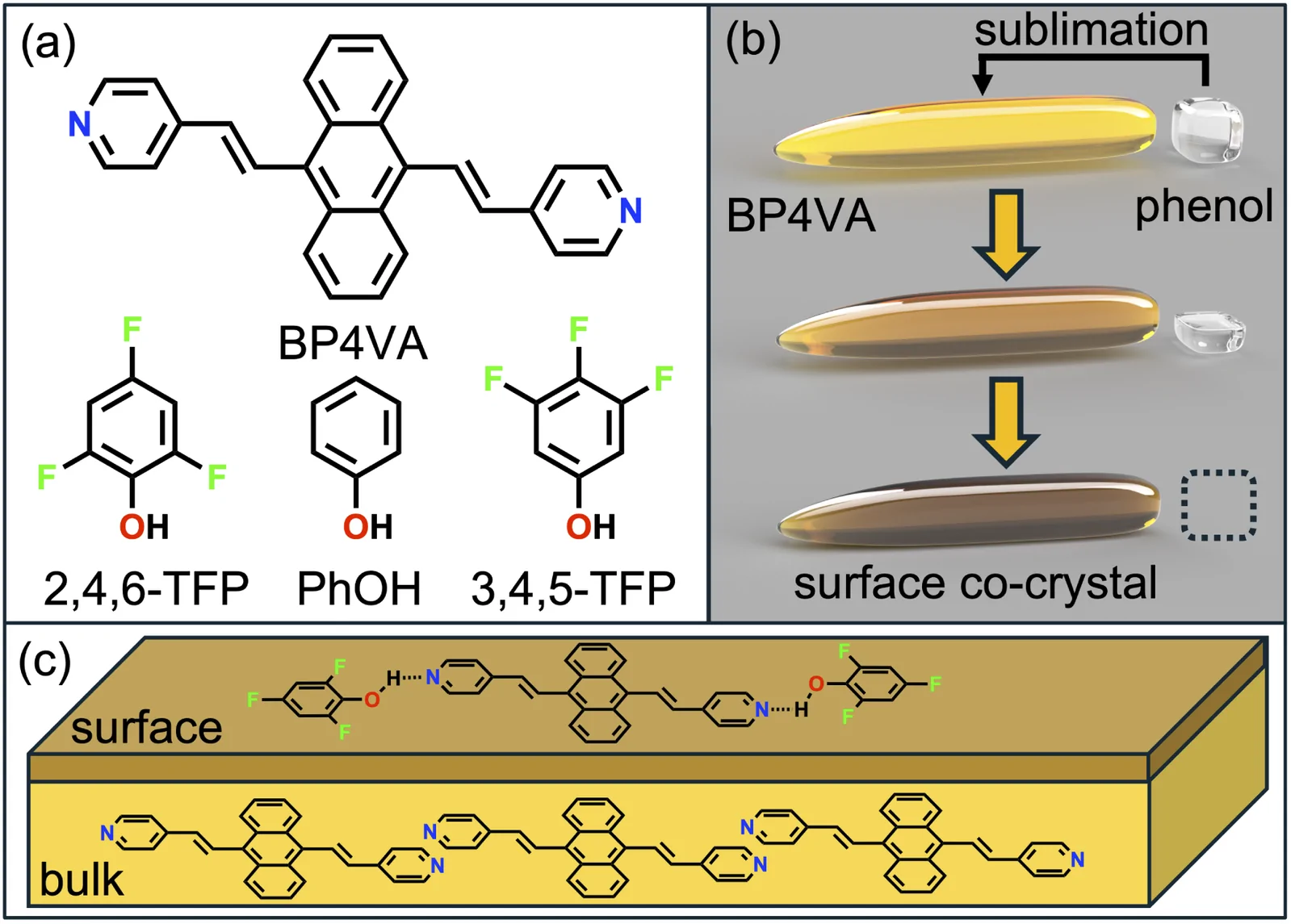
Detection of phenols using BP4VA single crystals: (a) molecular structures of BP4VA and the phenols used in this study. (b) Surface co-crystallization of BP4VA with phenol *via* vapor sublimation. (c) Surface co-crystallization on BP4VA crystals.

Here, we present the use of single crystals of 9,10-bis((*E*)-2-(pyridin-4-yl)vinyl)anthracene (BP4VA), an organic semiconductor (OSC) with chromic and luminescent properties,^10–13^ for the detection of vapors of 2,4,6-trifluorophenol (2,4,6-TFP), 3,4,5-trifluorophenol (3,4,5-TFP), and phenol (PhOH) which are solid VOCs that sublime at room temperature and pressure. Phenols have received widespread attention due to their observed cytotoxicity, developmental toxicity, growth-inhibiting properties, and their presence in drinking water in developed countries.^14–16^ Our detection method leverages the vapor exposure of solids 2,4,6-TFP, 3,4,5-TFP, and PhOH to single crystals of BP4VA with *in situ* detection performed using polarized optical microscopy (POM). Upon exposure, BP4VA crystals rapidly change color from bright yellow to dark orange, accompanied by decreased fluorescence intensity. Notably, this process is reversible *via* thermal annealing of the exposed BP4VA crystals. The detection mechanism is elucidated through a combination of single-crystal and powder X-ray diffraction (SCXRD and PXRD), Fourier-transform infrared spectroscopy (FT-IR), scanning electron microscopy (SEM), and energy dispersive X-ray spectroscopy (EDX). Our results demonstrate that phenol detection occurs at the surface of BP4VA crystals *via* co-crystallization mediated by [O–H⋯N] hydrogen bonding (Scheme 1). While molecular transformations on single-crystal metal surfaces have been well-documented,^24,25^ surface co-crystallization in organic single crystals for vapor detection of VOCs *via* sublimation is, to the best of our knowledge, unprecedented. We envisage vapor-driven surface co-crystallization as a promising strategy for molecular recognition and optical property modulation in organic crystalline materials.

## 2 Results and discussion

### 2.1 Design strategy and rationale for VOC detection

**Fig. 1:**
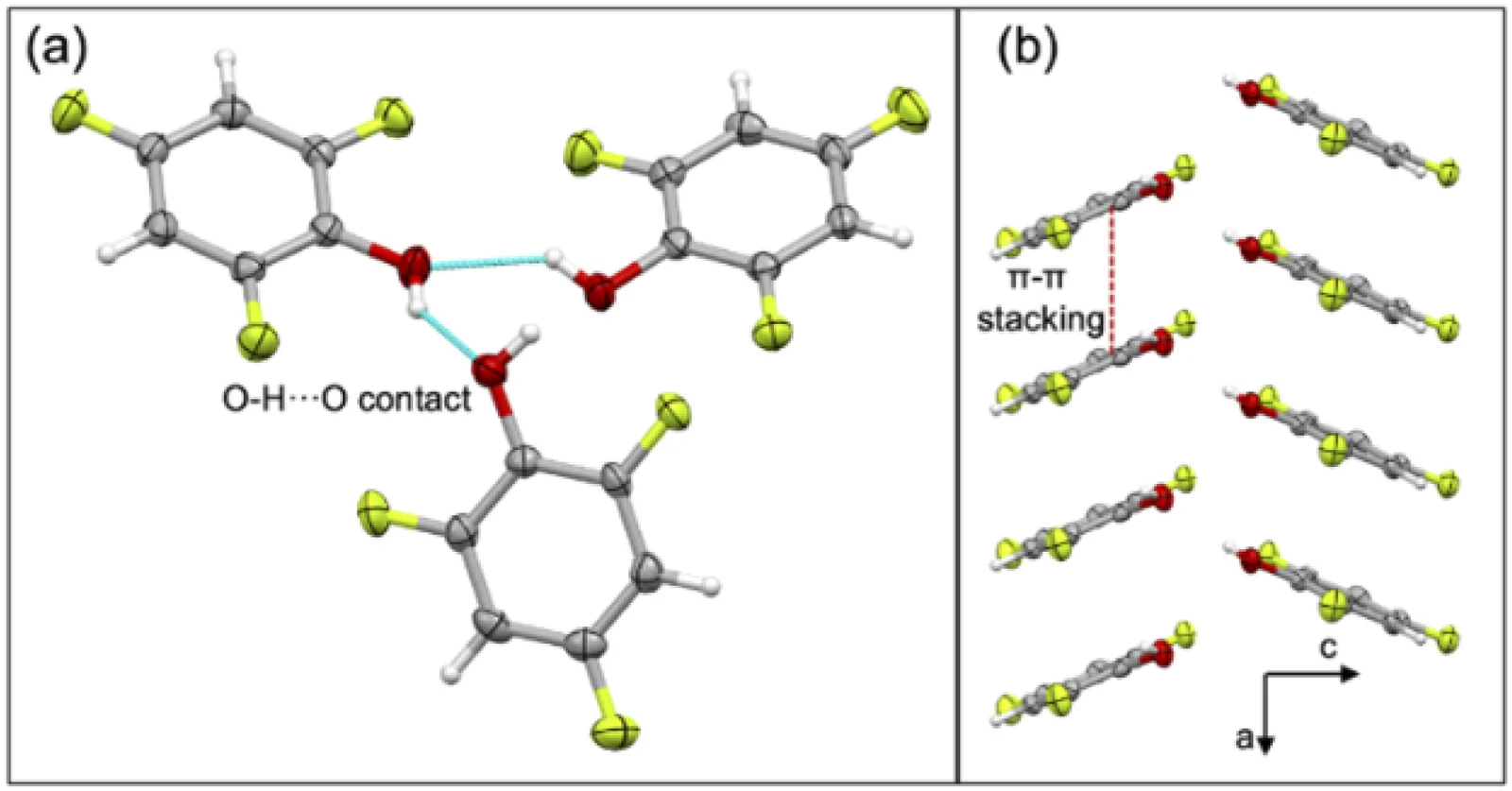
X-ray crystal structure of 2,4,6-TFP: (a) assembly of 2,4,6-TFP*via* [O–H⋯O] hydrogen bonding, and (b) columnar aggregation *via* [π–π] stacking along the *a*-axis.

Due to their small molecular weight and weak intermolecular contacts, phenols typically exhibit low sublimation and vaporization enthalpies,^26^ which, combined with their toxicity, make them a relevant target for vapochromic detection. PhOH, 2,4,6-TFP, and 3,4,5-TFP sublime readily at room temperature. To design a strategy for detecting phenols, a single-crystal X-ray diffraction (SCXRD) experiment was performed on crystals of 2,4,6-TFP. SCXRD revealed that 2,4,6-TFP molecules interact primarily *via* [O–H⋯O] hydrogen bonding with additional [π–π] stacking leading to columnar aggregation of 2,4,6-TFP along the *a*-axis, as illustrated in Fig. 1. A comparable stacking behavior was observed for collected crystals of 3,4,5-TFP (Fig. S3, SI). We envisage the use of suitable luminescent OSC crystals with accessible H-bond acceptors, such as BP4VA, could facilitate surface interaction with vapors of 2,4,6-TFP and related phenols *via* [O–H⋯N] contacts, leading to chromic and/or luminescent changes.

### 2.2 Detection of phenols with crystals of BP4VA

**Fig. 2:**
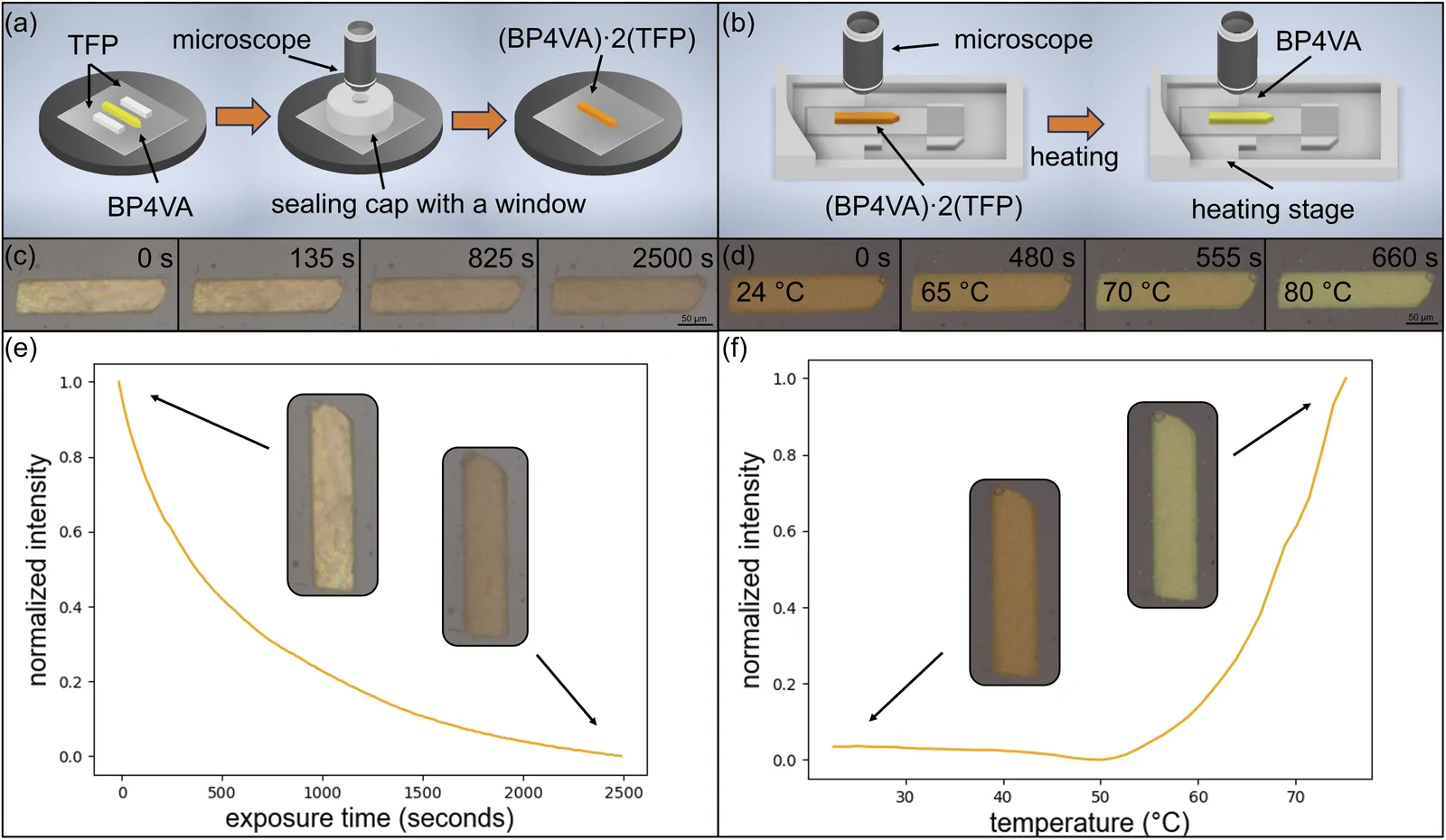
Vapochromic detection of 2,4,6-TFP with BP4VA crystals: (a) POM setup with a sealing cap as a closed chamber for exposure of BP4VA crystals to 2,4,6-TFP solids, and (b) resistive-heating thermal annealing of exposed BP4VA crystals. (c) Time-lapse of the vapochromic transformation of a BP4VA crystal upon exposure to 2,4,6-TFP solids, and (d) thermal annealing of exposed BP4VA crystals, with the corresponding normalized intensity *vs.* time plots (e and f).

To test our molecular crystal sensing platform, phenol detection with single crystals of BP4VA was evaluated by a series of *in situ* POM experiments carried out in a closed chamber (Fig. 2a). Exposure of BP4VA crystals to vapors of 2,4,6-TFP led to color change of the crystal surface, going from yellow (exposure time: 0 seconds) to dark orange (exposure time: 2500 seconds) (Fig. 2b and Video S1). To quantify the vapochromic change, the average intensity (mean gray value) of the region of interest (ROI) corresponding to the crystal at each timeframe has been calculated using ImageJ. Min–max normalization was employed to standardize data for analysis (Fig. 2c and e). Both quantitative and qualitative data show a significant difference in color intensity between BP4VA before and after exposure to 2,4,6-TFP. The vapor pressure of 2,4,6-TFP required for detection was estimated to be ≈1 mmHg using isothermal thermogravimetric analysis (Fig. S22, SI). The color change is reminiscent of photochromic crystals, which undergo intramolecular changes upon light irradiation.^27–29^ Notably, the exposed single crystals of BP4VA to vapors of 2,4,6-TFP solids were deemed recoverable after thermal annealing. Specifically, a Linkam LTS420 heating stage was used to heat the exposed BP4VA crystals (Fig. 2b and Video S3) using a ramp rate of 5 °C min^−1^ with a maximum temperature of 80 °C. The crystals showed a color change from dark orange to yellow and a gradual increase in intensity, as demonstrated in Fig. 2d and f, respectively.

**Fig. 3:**
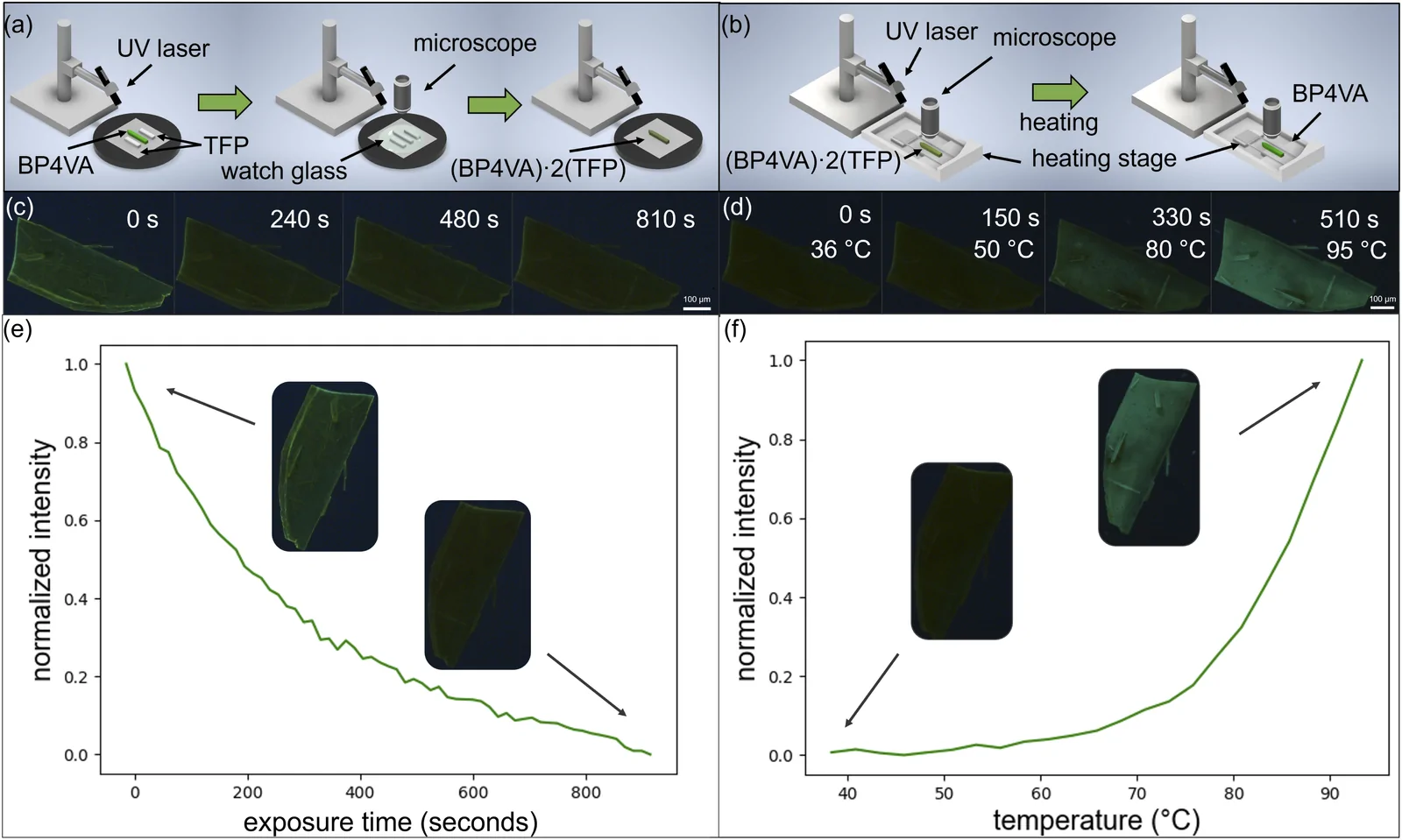
Luminescence intensity change of BP4VA crystals upon 2,4,6-TFP exposure: POM setup for luminescence monitoring upon (a) 2,4,6-TFP exposure of BP4VA crystals and (b) thermal annealing of exposed BP4VA crystals. Time-lapse of the luminescence intensity change of a BP4VA crystal during (c) exposure to 2,4,6-TFP vapor, and (d) thermal annealing of exposed crystals, with the corresponding normalized luminescence intensity *vs.* time plots (e and f).

Pristine crystals of BP4VA are fluorescent.^10–13^ We observed that exposure of BP4VA crystals to 2,4,6-TFP vapor decreased the fluorescence intensity in the visible spectrum using POM. Specifically, fluorescent BP4VA crystals (exposure time: 0 s) irradiated with a 405 nm class IIIA UV laser mounted on a stand (Fig. 3a) became de facto non-fluorescent at an exposure time of 810 s (Fig. 3c). Quantitatively, this change is described by a significant reduction in the intensity of the green channel (Fig. 3e and Video S2). Local absorption and photoluminescence (PL) spectra of BP4VA single crystals after 2,4,6-TFP exposure were taken to provide a rationale for luminescence quenching (Fig. S21, SI). Both spectra reveal significant red-shifts towards wavelengths at which the starting materials neither absorb nor emit.^10,11,30^ This shift indicates charge transfer (CT) state formation, suggesting co-crystallization as a driver for luminescence decrease.^11,31,32^ The exposed BP4VA crystals regained fluorescence upon thermal annealing. After being heated using the heating stage at a 10 °C min^−1^ rate to 95 °C, the exposed BP4VA crystals under a 405 nm class IIIA UV laser (Fig. 3b) showed a fluorescence recovery in the visible spectrum (Fig. 3d and Video S4). This recovery is quantified by the increase in the intensity of the green color channel (Fig. 3f). The single crystal sensing platform was found to be reusable with an approximate half-life of four exposure-annealing cycles (Fig. S7, SI). The potential for detecting other phenolic VOCs was further demonstrated by testing 3,4,5-TFP and PhOH solids, where BP4VA exhibited similar photochromic changes upon exposure to vapors *via* sublimation (Fig. S6–S8 and Videos S5–S10, SI).

### 2.3 X-ray diffraction and FT-IR characterizations of pristine and exposed BP4VA crystals

**Fig. 4:**
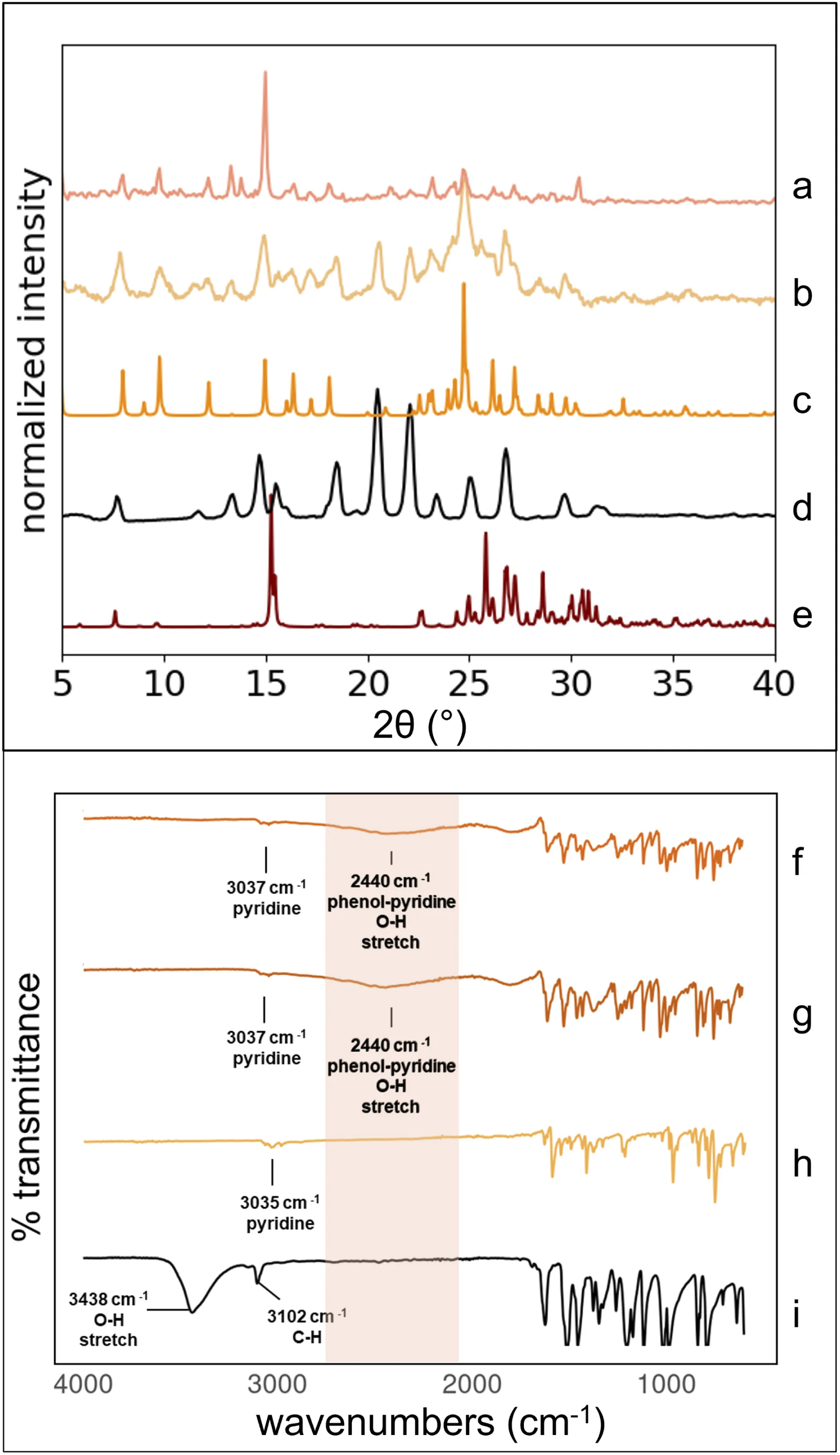
(Top) Powder X-ray diffraction traces of BP4VA·2(2,4,6-TFP) co-crystals prepared by (a) slow evaporation from solution, and (b) vapor exposure (sublimation), (c) calculated from single crystal data, (d) BP4VA experimental, (e) 2,4,6-TFP calculated from single crystal data. (Bottom) FT-IR spectra of BP4VA·2(2,4,6-TFP) co-crystals prepared by (f) slow evaporation from solution, (g) vapor exposure (sublimation), (h) BP4VA, and (i) 2,4,6-TFP starting material. Relevant peaks for co-crystal formation are highlighted.

A PXRD diffractogram of exposed BP4VA crystals with 2,4,6-TFP, solution-grown co-crystals (slow evaporation), and starting materials provided insight into the surface co-crystallization process (Fig. 4 Top). In particular, peaks below 2*θ* = 20° in the exposed crystals were assigned to a co-crystalline phase (*i.e.*, BP4VA·2(2,4,6-TFP), *vide infra*), while reflections of pristine BP4VA were also present (2*θ* = 20°, 23°). The difference between two phases was further confirmed by Scherrer analysis (SI). Single crystals examined by SCXRD consistently indexed to the unit cell of the *P*2_1_/*c* polymorph of BP4VA, indicating that, despite the observed vapochromic response upon exposure to 2,4,6-TFP, the crystals did not undergo complete crystal-to-amorphous or crystal-to-crystal transformations, as reported for other vapochromic materials exposed to solvent or liquid vapors.^17–23^ This lack of transformation is attributed to the closed-packed structure of the BP4VA crystal^10^ and the relatively large size of the 2,4,6-TFP molecule, which likely hinders guest transport through the crystal lattice. Instead, vapor exposure generates a polycrystalline co-crystal film of BP4VA·2(2,4,6-TFP, *vide infra*). We note that polycrystalline BP4VA powder is also able to sustain the formation of BP4VA·2(2,4,6-TFP), alongside corresponding photochromic changes.

**Fig. 5:**
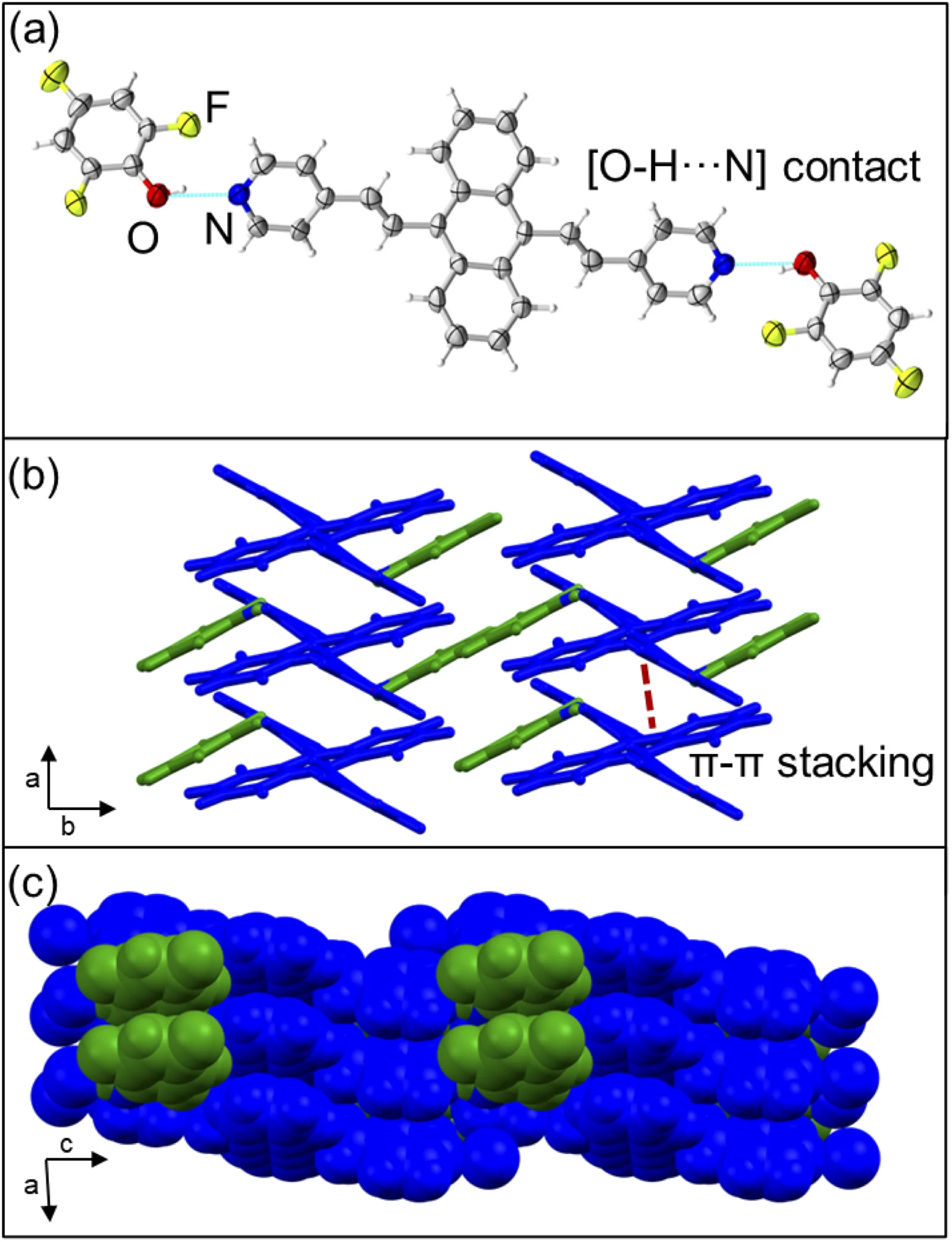
X-ray crystal structure of BP4VA·2(2,4,6-TFP): (a) three-component assembly sustained by [O–H⋯N] hydrogen bonds, (b) corrugated [π⋯π] assemblies in the *ab*-plane, and (c) space-filling view of corrugated sheets along the *c*-axis.

To gain deeper insight into the interaction between BP4VA and TFPs, single crystals of BP4VA (15 mg, 0.039 mmol) with 2,4,6-TFP (11.6 mg, 0.078 mmol) were grown as orange prisms by slow evaporation of a 2 : 1 (v/v) chloroform/acetonitrile solution (3 mL). ^1^H nuclear magnetic resonance (NMR) spectroscopy confirmed the stoichiometry of the crystals to be BP4VA·2(2,4,6-TFP) (Fig. S15, SI). In contrast, vapor-exposed crystals exhibited a higher BP4VA-to-TFP ratio, further supporting a surface-limited co-crystallization process (Fig. S17, SI). A SCXRD analysis of BP4VA·2(2,4,6-TFP) revealed the components to crystallize in the triclinic space group *P*1̄ as a three-component assembly (*i.e.*, co-crystal), where a unit of BP4VA bridges two 2,4,6-TFP molecules *via* [O–H⋯N] H-bonds (1.83 Å) (Fig. 5a). The H-bonded assembly is comparable to reports of co-crystals with bipyridines.^33–37^ The presence of a [O–H⋯N] H-bond in BP4VA·2(2,4,6-TFP) was confirmed with FT-IR spectroscopy, which showed the disappearance of the pristine phenol O–H stretch of 2,4,6-TFP at ≈3438 cm^−1^ and new bands to appear at ≈2440 cm^−1^, which are in agreement with an O–H stretching in a phenol–pyridine in a co-crystal (Fig. 4 bottom).^38,39^

The anthracene core in adjacent assemblies in the *ab*-plane exhibits offset face-to-face [π⋯π] contacts (Fig. 5b). Extended packing shows the supramolecular aggregates organized in corrugated sheets along the *c*-axis (Fig. 5c). Notably, the previously unidentified PXRD peaks of exposed BP4VA crystals closely matched the simulated PXRD pattern from BP4VA·2(2,4,6-TFP). Based on this observation, we hypothesize that BP4VA interaction with 2,4,6-TFP*via* vapor exposure takes place solely on the surface of the crystals (*i.e.*, surface co-crystallization). Generality of the surface co-crystallization approach of BP4VA was observed with 3,4,5-TFP and PhOH, which generated BP4VA·2(3,4,5-TFP) and BP4VA·2(PhOH) co-crystals as determined by SCXRD, PXRD, FT-IR and ^1^H NMR data (Fig. S1, S2, S4, S5, S10, S11, S16 and S18, SI) by vapor exposure (*i.e.*, sublimation) and slow evaporation methods.

### 2.4 SEM and EDX characterization of surface morphology and chemical composition

**Fig. 6:**
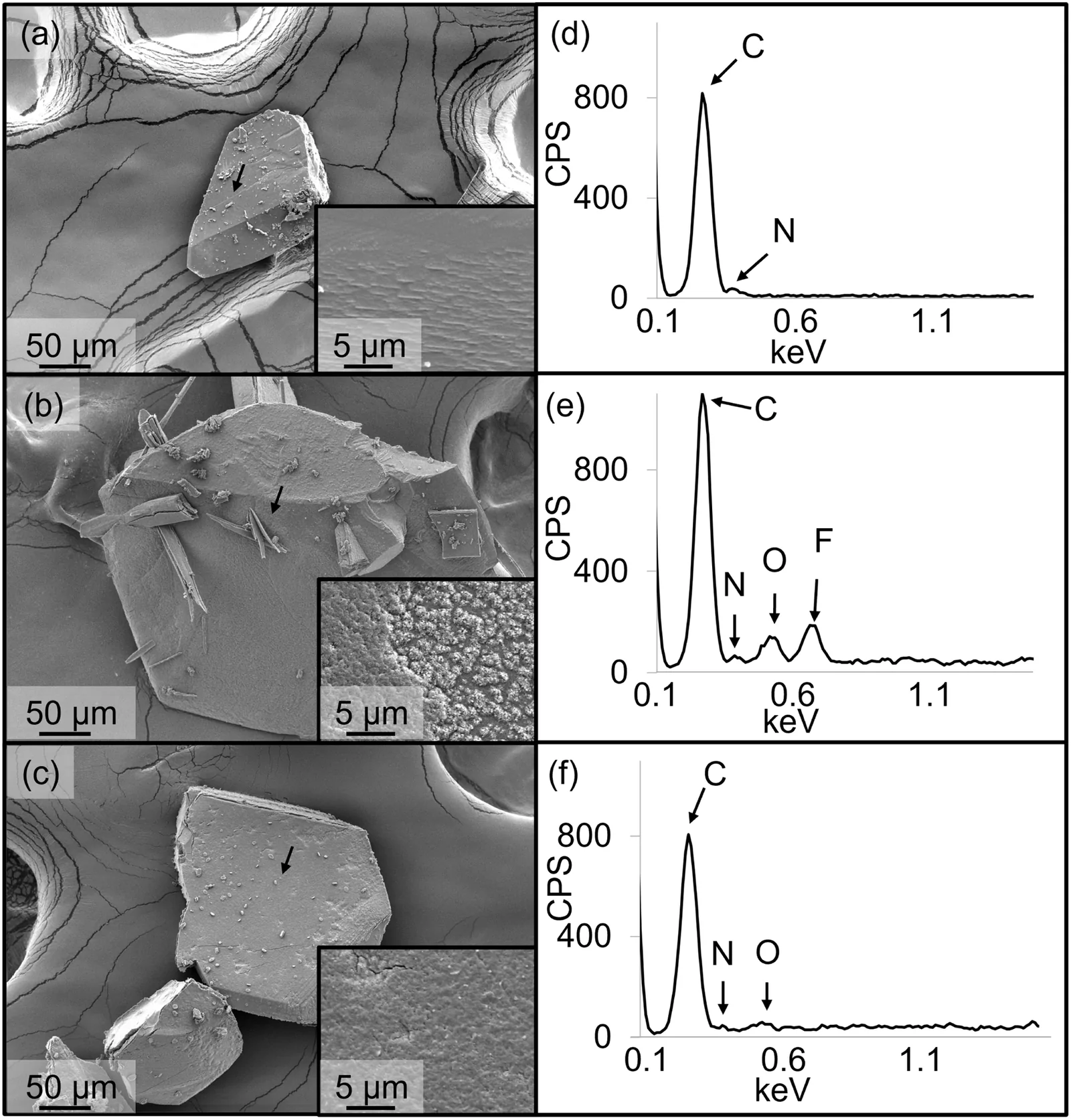
SEM images and EDX spectra of BP4VA exposed to 2,4,6-TFP. (a) SEM image of pristine BP4VA, (b) after exposure to 2,4,6-TFP and (c) after annealing at 80 °C to recover the original crystal. (d) EDX spectrum of pristine BP4VA, (e) after exposure to 2,4,6-TFP and (f) after annealing at 80 °C to remove 2,4,6-TFP. The inset high magnification images were taken from the location indicated by the arrow in each low magnification image.

The reversibility of surface morphology and composition changes in BP4VA crystals after exposure to the TFPs was characterized using SEM and EDX. The SEM images were taken at the same magnifications to show the range of crystal sizes and surface textures. Most single crystals were around 100 to 500 µm in size, with some smaller crystallites (∼2–10 µm) present on the surfaces of the larger crystals. Fig. 6a shows that before exposure, the pristine crystals exhibit a smooth surface morphology, visible in the high-magnification inset image (inset images are from locations indicated by black arrows on each crystal). After exposure to 2,4,6-TFP, the crystal surface transforms into a porous, highly textured structure with clustered, dendritic formations, as detailed in the inset image (Fig. 6b). The depth of the transformation was estimated to equal 1.5 µm *via* cross-sectional SEM (Fig. S20, SI). Remarkably, the surface transformations show strong signs of reversibility through low-temperature annealing (80 °C). Fig. 6c demonstrates that annealing the exposed crystals at 80 °C for approximately 11 minutes restores much of the original smooth surface morphology, though some dendritic clusters persist (shown in the inset high-magnification image). Although some areas of the annealed crystals still show morphological changes from 2,4,6-TFP exposure, the tendency toward significant reversal of the surface morphology is promising for sensing applications.

Furthermore, EDX elemental analysis confirms the removal of oxygen (O) and fluorine (F) from BP4VA exposed to 2,4,6-TFP after annealing. The pristine BP4VA spectrum in Fig. 6d has a strong carbon (C) peak and a smaller nitrogen (N) peak (although the N Kα peak is typically affected by significant absorption, it is visible in the EDX spectra in Fig. 6d–f due to its relatively high concentration in the compound). After exposure to 2,4,6-TFP (Fig. 6e), significant O and F peaks appear as a result of surface co-crystallization. Annealing reduces the relative intensity of the O and F peaks (Fig. 6f). Similar behavior was observed for BP4VA exposed to 3,4,5-TFP (Fig. S19, SI). We note that crystals exposed to 3,4,5-TFP left under ambient conditions for five months also showed a tendency to reverse to a near-pristine surface morphology (Fig. S19d, SI). These SEM and EDX results support the reversibility phenomenon demonstrated by the vapochromic and luminescence results, highlighting BP4VA's potential as a reusable OSC crystal for VOC detection.

## 3 Conclusion

In conclusion, we have demonstrated the vapochromic and luminescent detection of vapor from solid phenol samples using single crystals of BP4VA*via* surface co-crystallization. Specifically, POM studies revealed significant changes in both color and luminescence intensities of BP4VA upon exposure to phenols in a closed chamber. SCXRD, PXRD and FT-IR revealed that the surface interaction was primarily facilitated by [O–H⋯N] hydrogen bonding. Notably, POM studies showed the exposed BP4VA crystal to have a tendency toward reversibility, with the original lighter coloration restored naturally by aging, or accelerated by thermal annealing. The reversibility was further supported by changes in surface morphology, as observed in SEM images, and by the observation of O and F characteristic X-ray peaks in EDX spectra. To the best of our knowledge, surface co-crystallization *via* sublimation on an organic single crystal and its application in molecular detection are unprecedented. These findings suggest that the stimuli-responsiveness and recoverability of BP4VA make it and related OSCs promising candidates for the detection of small organic molecules *via* hydrogen bonding and other non-covalent interactions. We are currently advancing the design of single-crystal-based sensors that integrate additional attributes (*e.g.*, mechanical compliance, conductivity), to expand the scope and tunability of surface co-crystallization-based detection.

## Author contributions

I. B. and K. H.: carried out investigation, methodology, curation, validation and writing the original draft. E. J. C.-V., A. D., A. S., J. B., S. J. T. and N. S. S.: carried out investigation, formal analysis, methodology, data curation, and validation. A. M. G., J. J., S. S. L.: performed formal analysis, data curation, and review & editing of the original draft. G. C.-A.: contributed by formal analysis, conceptualization, project administration, funding acquisition, and writing and reviewing the original draft.

## Conflicts of interest

There are no conflicts to declare.

## Supplementary Material

SC-017-D6SC05755A-s001

SC-017-D6SC05755A-s002

SC-017-D6SC05755A-s003

SC-017-D6SC05755A-s004

SC-017-D6SC05755A-s005

SC-017-D6SC05755A-s006

SC-017-D6SC05755A-s007

SC-017-D6SC05755A-s008

SC-017-D6SC05755A-s009

SC-017-D6SC05755A-s010

SC-017-D6SC05755A-s011

SC-017-D6SC05755A-s012

SC-017-D6SC05755A-s013

## Data Availability

CCDC 2420836–2420838 (2,4,6-TFP), BP4VA·2(2,4,6-TFP), BP4VA·2(3,4,5-TFP), 2488710 (3,4,5-TFP) and 2571288 (BP4VA·2(PhOH)) contain the supplementary crystallographic data for this paper.^40*a*–*e*^ The authors confirm that the data supporting the findings of this study are available within the article and its supplementary information (SI). Supplementary information: experimental information, FT-IR, NMR, PXRD patterns, absorption and emission spectra, SEM micrographs, additional SCXRD and POM data. See DOI: https://doi.org/10.1039/d6sc05755a.
