# Supplementary material for: Surface co-crystallization with an organic single crystal enables vapochromic and luminescent detection of phenols
Source: Chem Sci. 2026 Aug 18;17(34):16163–9. doi: 10.1039/d6sc05755a (PMC13482463; doi:10.1039/d6sc05755a)
Supplement: Supplementary file 1 [file SC-017-D6SC05755A-s001.pdf]

Supplementary Information (SI) for Chemical Science.
This journal is © The Royal Society of Chemistry 2026

Supplementary Information (SI)

Surface co-crystallization with an organic single crystal enables vapochromic and luminescent detection of phenols

Ivan Bondarenko,† Kaleb Hood,‡ Elani J. Cabrera-Vega,¶ Aminata Dioume,§ Alaric Shaw,† Josephine Bicknell,† Simon J. Teat,‖ Nicholas S. Settineri,‖ ,⊥ Andrea M. Goforth,¶ Stephanie S. Lee,§ Jun Jiao,‡ and Gonzalo Campillo-Alvarado*,†

†*Department of Chemistry, Reed College, Portland, OR, United States*

‡*Department of Mechanical and Materials Engineering, Portland State University, Portland, OR, United States*

¶*Department of Chemistry, Portland State University, Portland, OR, United States*

§*Molecular Design Institute, Department of Chemistry, New York University, New York, New York 10003, United States.*

‖*Advanced Light Source, Lawrence Berkeley National Laboratory, Berkeley, CA, 94720, United States*

⊥*Department of Chemistry, University of California, Berkeley, Berkeley, California, 94720-1460, United States*

E-mail: gcampillo@reed.edu

S1 Experimental information

Materials and Synthesis

Materials. Acetonitrile, DCM, and chloroform were purchased from Sigma–Aldrich. **BP4VA** was purchased from AChemBlock and AmBeed. **2,4,6-TFP** and **3,4,5-TFP** were purchased from AmBeed. **PhOH** was purchased from TCI. All the solvents and reagents were used without further purification.

Synthesis of BP4VA·2(2,4,6–TFP). BP4VA (15 mg, 0.039 mmol) and **2,4,6–TFP** (11.6 mg, 0.078 mmol) were dissolved in 3 mL of a mixture of chloroform and acetonitrile (2:1 v/v ratio) with the aid of heat and sonication After three days of slow evaporation, suitable crystals for single crystal X–ray diffraction (SCXRD) were collected.

Synthesis of BP4VA·2(3,4,5–TFP). BP4VA (15 mg, 0.039 mmol) and **3,4,5-TFP** (11.6 mg, 0.078 mmol) were dissolved in 3 mL of chloroform with the aid of heat and sonication. After three days of slow evaporation, suitable crystals for single–crystal X–ray diffraction (SCXRD) were collected.

Synthesis of BP4VA·2(PhOH). BP4VA (15 mg, 0.039 mmol) and **PhOH** (7.3 mg, 0.078 mmol) were dissolved in 3 mL of DCM with the aid of heat and sonication. After five months of slow evaporation in the fridge, suitable crystals for single–crystal X–ray diffraction (SCXRD) were collected.

Crystallization of BP4VA. BP4VA (15 mg, 0.039 mmol) was dissolved in 3 mL of a mixture of chloroform and acetonitrile (2:1 v/v ratio) with the aid of heat and sonication. After three days of slow evaporation, suitable crystals for POM were collected. The unit cell of the crystals corresponded to the $P2_1/c$ polymorph.[1]

Instruments and Methods

Single–crystal X–ray diffraction (SCXRD) data for all structures except **3,4,5-TFP** and **BP4VA·2(PhOH)** were collected on a Rigaku XtaLAB Mini·II diffractometer with a CCD

area detector (λMoKα=0.71073 Å, monochromator: graphite). Experiments were conducted at room temperature. The collected data was refined with CrysAlisPro through standard data reduction and background corrections (multiscan for all crystals). The **3,4,5-TFP** structure was collected at 100 K using a Bruker D8 Venture IμS microfocus dual-source diffractometer equipped with a PHOTON II CPAD detector (CuKα λ= 1.54178 Å) and an Oxford cryogenic 800 system. Cell measurement, data collection, integration, scaling, and absorption correction were achieved using the APEX4 software. Data were processed using SAINT, and an absorption correction was performed using SADABS programs incorporated in APEX. For both set-ups, the crystals were mounted in Paratone oil on a Mitegen magnetic mount. SCXRD data for **BP4VA·2(PhOH)** were collected on beamline 12.2.1 at Advanced Light Source. The intensity data with synchrotron radiation were collected in APEX3, integration and corrections were applied with SAINT v8.40b, and absorption and other corrections were made using SADABS 2016/2. Dispersion corrections appropriate for this wavelength were calculated using the Brennan method in XDISP4[2] within WinGX.[3] All non-hydrogen atoms were refined anisotropically. Hydrogen atoms were placed geometrically on the carbon atoms and refined with a riding model. Structure solution and refinement were performed using SHELXT[4] and SHELXL,[5] respectively, within the Olex2[6] graphical user interface. Crystal structures were generated using Mercury.

Rigaku Ultima IV X–ray diffraction system (Tokyo, Japan) using a parallel beam alignment with graphite monochromatized CuKα radiation was used to collect PXRD spectra for all systems except **BP4VA·2(PhOH)**. The samples were mounted and collected on zero–background (Si (100)) micro–holder slides in the range of 0 to 40° 2θ (scan type: step size: 0.1°, rate: 3 deg/min). The equipment was operated at room temperature. The resulting data was analyzed using Rigaku PDXL2 software and plotted in Python. The data for **BPVA**·2(**PhOH**) was collected on a Scintag XDS-2000 diffractometer using CuKα1 radiation (λ = 1.5418 Å). The samples were mounted and collected on glass slides in the range of 5–40° two-theta (scan type: step size: 0.02°, rate: 3 deg/min, continuous scan mode). The

equipment was operated at 40 kV and 30 mA, and data were collected at room temperature.

Fourier–transform infrared spectroscopy (FT–IR) spectra were recorded on a Thermo Scientific Nicolet iS5 FT–IR spectrometer from 600 to 4000 cm^{-1} with an iD5 attenuated total reflectance (ATR) accessory. Crystalline samples were used for each experiment. FT–IR data were visualized with RStudio, and peak analysis was conducted in SpectraGryph software. ^{1}H NMR spectra were recorded on a Bruker Ascend Evo 400 MHz spectrometer with DMSO–d_6 as internal standard. For experiments on vapor-induced surface co-crystals, a batches of **BP4VA** single crystals with an approximate average surface area 0.01 – 0.02 mm^2 were used.

Polarized optical microscopy data was collected with a Nikon ECLIPSE Ci–POL polarized light microscope under 10× magnification. For every experiment, a suitable **BP4VA** single crystal was placed on a glass slide, and approximately 85 mg of **2,4,6-TFP**,**3,4,5-TFP** or **PhOH** was placed next to the crystal. Either a 20 mL vial cap with a 0.17 mm glass window (for chromodetection experiments) or a watch glass (for fluorescence–based detection experiments) was placed on top of the slides to prevent phenol vapor from escaping. Microscope illumination was utilized as the only light source for chromodetection experiments and a 405 nm class IIIA UV laser mounted on a stand was utilized as the only light source for fluorescence–based detection experiments. For the thermal recovery experiments, a Linkam LTS420 heating stage with XY manipulation was used to heat the exposed **BP4VA** crystals. The temperature ramp was set to 5 °C min^{-1} with a maximum temperature of 80 °C for chromodetection experiments and to 10 °C min^{-1} ramp rate with a maximum temperature of 100 °C for fluorescence–based detection. ImageJ software was used to extract the average intensity (mean gray value) of a representative region of interest (ROI) containing the single crystal using a multi–measure tool. Min–max normalization was then performed via Python for the data analysis. For the cycling experiments, the standard chromodetection and thermal recovery procedure was performed on the same crystal four times. ImageJ software was used to extract the average intensity of a representative ROI of the snapshots of a

single crystal before and after exposure for each cycle. The relative intensity decrease was calculated *via* the (intensity before − intensity after)/(intesnity before) × 100 formula.

Scanning electron microscope (SEM) images and energy dispersive X–ray (EDX) spectra were acquired on an SNE 4500M Plus SEM with an Oxford Compact EDX detector operating at a 5 keV accelerating voltage. A thin gold coating was sputtered on the crystals to prevent charging, which had no effect on surface structures compared to uncoated crystals.

Local absorption and photoluminescence (PL) spectra were collected on 50x50 μm^2 and 70x70 μm^2 areas, respectively, of each crystal using a CRAIC 508PV^{TM} microspectrophotometer mounted on a microscope.

Isothermal thermogravimetric analysis (TGA) was performed on a 2,4,6-**TFP** sample using a TA Instrument TGA Q50 operating under a nitrogen atmosphere (40 mL/min) with a platinum pan at 22.6 °C.

S2 Single-crystal X-ray data

Crystallographic tables

Table S1: Crystallographic parameters for **BP4VA**·2**(3,4,5–TFP)**

Parameter	Value
Compound name	**BP4VA**·2**(3,4,5–TFP)**
Empirical formula	$C_{20}H_{13}F_3NO$
Formula weight	340.31
Temperature / K	298(2)
Crystal system	triclinic
Space group	P-1
a/Å	5.0529(3)
b/Å	10.9780(7)
c/Å	15.1080(7)
$\alpha/^\circ$	104.010(4)
$\beta/^\circ$	99.606(4)
$\gamma/^\circ$	94.208(4)
Volume / Å^3	796.03(8)
Z	2
$\rho_{\text{calc}}/\text{g cm}^{-3}$	1.420
μ/mm^{-1}	0.112
$F(000)$	350.0
Crystal size	$0.39 \times 0.21 \times 0.15$ mm^3
Radiation	Mo Kα (λ = 0.71073 Å)
2Θ range / °	4.148–49.996
Index ranges	$-5 \leq h \leq 6,\ -13 \leq k \leq 12,\ -17 \leq l \leq 17$
Reflections collected	8644
Independent reflections	2770 [R_{int}=0.0366, R_{sigma}=0.0312]
Data/restraints/parameters	2770/37/259
Goodness-of-fit on F^2	1.130
Final R [I$\geq 2\sigma(I)$]	R_1=0.0527, wR_2=0.1627
Final R [all data]	R_1=0.0731, wR_2=0.1725
CCDC ID	2420838

Table S2: Crystallographic parameters for **BP4VA**·2**(2,4,6–TFP)**

Parameter	Value
Compound name	**BP4VA**·2**(2,4,6–TFP)**
Empirical formula	$C_{20}H_{13}F_3NO$
Formula weight	340.31
Temperature / K	283(15)
Crystal system	triclinic
Space group	P-1
a/Å	3.9417(6)
b/Å	11.0938(18)
c/Å	17.808(2)
$\alpha/^\circ$	84.765(12)
$\beta/^\circ$	89.007(12)
$\gamma/^\circ$	87.456(13)
Volume / Å^3	774.6(2)
Z	2
$\rho_{\mathrm{calc}}/\mathrm{g\,cm^{-3}}$	1.459
$\mu/\mathrm{mm^{-1}}$	0.115
$F(000)$	350.0
Crystal size	0.22 × 0.06 × 0.05 mm^3
Radiation	Mo Kα (λ = 0.71073 Å)
2Θ range / °	4.166–49.998
Index ranges	$-4 \leq h \leq 4,\ -13 \leq k \leq 13,\ -21 \leq l \leq 21$
Reflections collected	10002
Independent reflections	2723 [R_{int}=0.1325, R_{sigma}=0.2115]
Data/restraints/parameters	2723/0/226
Goodness-of-fit on F^2	0.874
Final R [I≥ $2\sigma(I)$]	R_1=0.0791, wR_2=0.1352
Final R [all data]	R_1=0.2574, wR_2=0.1707
CCDC ID	2420837

Table S3: Crystallographic parameters for **BP4VA**·2**(PhOH)**

Parameter	Value
Compound name	**BP4VA**·2**(PhOH)**
Empirical formula	$C_{40}H_{32}N_2O_2$
Formula weight	572.67
Temperature / K	100(2)
Crystal system	monoclinic
Space group	$P2_1/c$
a/Å	5.8526(5)
b/Å	15.4894(13)
c/Å	16.2612(14)
α/°	90
β/°	95.065(3)
γ/°	90
Volume / Å^3	1468.4(2)
Z	2
ρ_{calc}/g cm^{-3}	1.295
μ/mm^{-1}	0.083
F(000)	604
Crystal size	0.250 × 0.020 × 0.015 mm^3
Radiation	synchrotron (λ = 0.7288 Å)
2Θ range / °	3.730–68.344
Index ranges	$-8 \leq h \leq 9,\ -23 \leq k \leq 23,\ -24 \leq l \leq 25$
Reflections collected	28569
Independent reflections	5571 [R_{int}=0.0571, R_{sigma}=0.0428]
Data/restraints/parameters	5571/0/203
Goodness-of-fit on F^2	1.068
Final R [I≥ $2\sigma(I)$]	R_1=0.0440, wR_2=0.1227
Final R [all data]	R_1=0.0505, wR_2=0.1284
CCDC ID	2571288

Table S4: Crystallographic parameters for **2,4,6-TFP**

Parameter	Value
Compound name	**2,4,6-TFP**
Empirical formula	$C_6H_3F_3O$
Formula weight	148.08
Temperature / K	99.8(9)
Crystal system	monoclinic
Space group	$P2_1/n$
a/Å	30.3379(17)
b/Å	3.6732(3)
c/Å	30.7596(19)
$\alpha/°$	90
$\beta/°$	98.660(6)
$\gamma/°$	90
Volume / Å^3	3388.7(4)
Z	24
$\rho_{calc}/g\,cm^{-3}$	1.742
μ/mm^{-1}	0.183
$F(000)$	1776.0
Crystal size	0.89 × 0.16 × 0.12 mm^3
Radiation	Mo Kα (λ = 0.71073 Å)
2Θ range / °	4.044–50.000
Index ranges	$-36 \leq h \leq 35,\ -4 \leq k \leq 4,\ -36 \leq l \leq 35$
Reflections collected	21662
Independent reflections	5981 [R_{int}=0.1427, R_{sigma}=0.0884]
Data/restraints/parameters	5981/0/487
Goodness-of-fit on F^2	1.091
Final R [I≥ $2\sigma(I)$]	R_1=0.0820, wR_2=0.2161
Final R [all data]	R_1=0.1108, wR_2=0.2431
CCDC ID	2420836

Table S5: Crystallographic parameters for **3,4,5-TFP**

Parameter	Value
Compound name	**3,4,5-TFP**
Empirical formula	$C_{12}H_6F_6O_2$
Formula weight	296.17
Temperature / K	293(2)
Crystal system	monoclinic
Space group	$P2_1/c$
a/Å	8.2993(15)
b/Å	20.050(3)
c/Å	6.9912(11)
$\alpha/°$	90
$\beta/°$	97.599(10)
$\gamma/°$	90
Volume / $Å^3$	1153.2(3)
Z	4
$\rho_{calc}/g\,cm^{-3}$	1.706
μ/mm^{-1}	1.623
$F(000)$	592.0
Crystal size	$0.16 \times 0.15 \times 0.1$ mm^3
Radiation	Cu Kα (λ = 1.54184 Å)
2Θ range / °	8.82–144.454
Index ranges	$-9 \leq h \leq 10,\ -24 \leq k \leq 24,\ -8 \leq l \leq 8$
Reflections collected	16080
Independent reflections	2272 [R_{int}=0.0384, R_{sigma}=0.0217]
Data/restraints/parameters	2272/0/185
Goodness-of-fit on F^2	1.054
Final R [I$\geq 2\sigma(I)$]	R_1=0.0356, wR_2=0.0863
Final R [all data]	R_1=0.0430, wR_2=0.0913
CCDC ID	2488710

Table S6: Selected hydrogen bonds and interactions in co-crystals

Crystal/parameter	d(F···H)	d(N···H)	d(C–H···π)	d(O···H)	$d(\pi\cdots\pi)$	Symm. code
**BP4VA**·2(**3,4,5-TFP**)	3.214(3)[1]	–	–	–	–	$(-X, -Y, 1-Z)$
	–	2.729(9)[2]	–	–	–	$(3-X, 1-Y, 2-Z)$
	–	–	4.191(3)[3]	–	–	$(1-X, 1-Y, 1-Z)$
	–	–	–	3.501(5)[4]	–	$(1-X, 1-Y, 1-Z)$
**BP4VA**·2(**2,4,6-TFP**)	3.355(6)[5]	–	–	–	–	$(-X, -Y, -Z)$
	3.564(7)[6]	–	–	–	–	$(-1+X, +Y, +Z)$
	–	2.640(5)[7]	–	–	–	$(-X, 1-Y, -1-Z)$
	–	–	3.563(6)[8]	–	–	$(-1+X, +Y, +Z)$
	–	–	–	3.269(7)[9]	–	$(1-X, -Y, -Z)$
	–	–	–	–	3.9417(6)[10]	$(-1-X, 1-Y, -1-Z)$
**BP4VA**·2(**PhOH**)	–	1.836(18)[11]	–	–	–	(X, Y, Z)
	–	–	2.9429(4)[12]	–	–	$(-1+X, Y, Z)$
	–	–	–	2.6164(7)[13]	–	$(1+X, Y, Z)$
	–	–	–	–	5.8526(5)[14]	$(-1+X, Y, Z)$

[1]C10–H10···F2; [2]O1–H1···N1; [3]C13–H13···π (centroid C9–C14); [4]C4–H4···O1; [5]C14–H14···F2; [6]C1–H1···F1; [7]O1–H1···N; [8]C1–H1···π (centroid N1–C5); [9]C5–H5···O; [10]$\pi\cdots\pi$ (centroid-to-centroid N1–C5); [11]O1–H···N1; [12]C7–H···π (centroid C9–C14); [13]C5–H5···O1; [14]$\pi\cdots\pi$ (centroid-to-centroid N1–C5).

BP4VA·2(3,4,5-TFP) X-ray Structure

Components in **BP4VA**·2(**3,4,5-TFP**) crystallize in triclinic space group P-1 with an asymmetric unit that contains one **3,4,5-TFP** unit linked with one-half of a **BP4VA** molecule. The grown structure shows three component assemblies where a unit of **BP4VA** bridges two **3,4,5-TFP** molecules via [O-H$\cdots$N] H-bonds (1.73 Å) (Figure S1a) The anthracene exhibits offset face-to-face [$\pi\cdots\pi$] contacts with adjacent assemblies (Figure S1b) An extended view of the supramolecular aggregates shows the solid to be close-packed with almost no voids present. The interactions organize aggregates in corrugated sheets along the c-axis (Figure S1c).

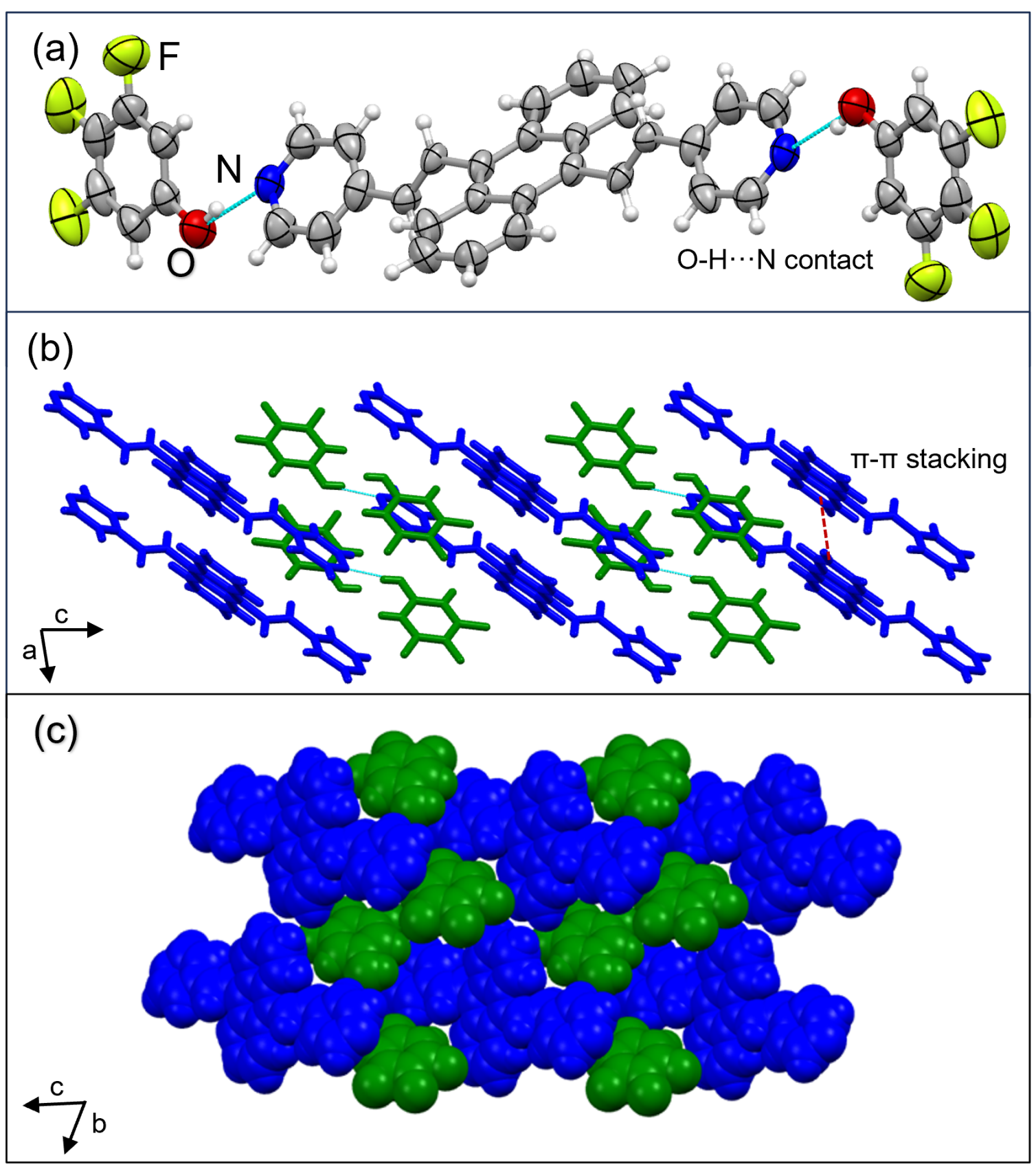

Figure S1: X-ray crystal structure of **BP4VA**·2(**3,4,5-TFP**): (a) three-component assembly sustained by [O–H$\cdots$N] hydrogen bonds, (b) corrugated [$\pi\cdots\pi$] assemblies in the ac-plane, and (c) space-filling view of corrugated sheets along the c-axis.

BP4VA·2(PhOH) X-ray Structure

Components in **BP4VA**·2**PhOH** crystallize in triclinic space group $P2_1/c$ with an asymmetric unit that contains one **PhOH** unit linked with one-half of a **BP4VA** molecule. The grown structure shows three component assemblies where a unit of **BP4VA** bridges two **PhOH** molecules via [O-H$\cdots$N] H-bonds (1.83 $\AA$) (Figure S2a) The anthracene exhibits offset face-to-face [$\pi\cdots\pi$] contacts with adjacent assemblies (Figure S2b) An extended view of the supramolecular aggregates shows the solid to be close-packed with almost no voids present. The interactions organize aggregates in corrugated sheets along the b-axis (Figure S1c).

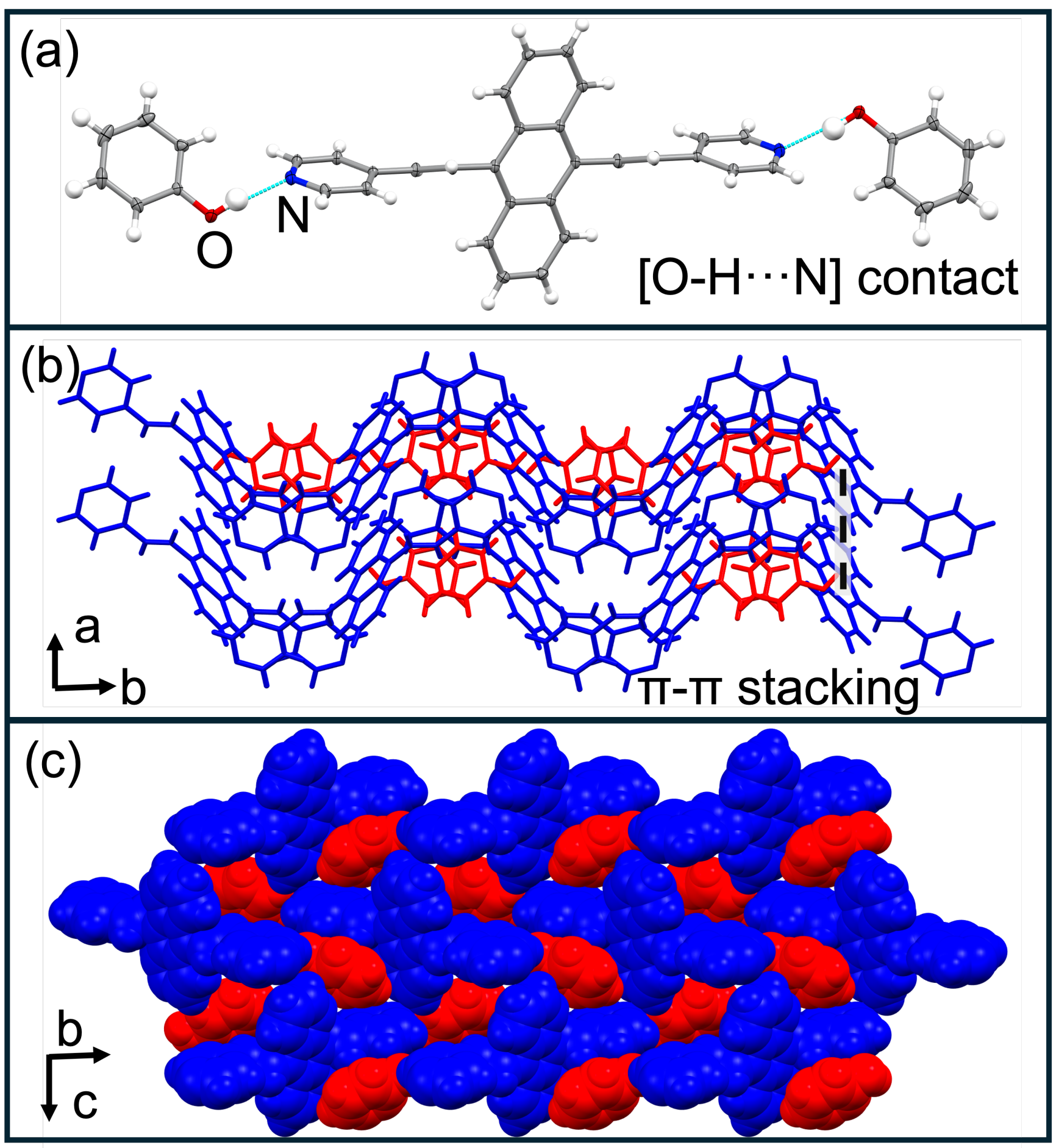

Figure S2: X-ray structure of **BP4VA·PhOH**:(a) three-component assembly sustained by [O–H$\cdots$N] hydrogen bonds, (b) corrugated [$\pi\cdots\pi$] assemblies in the ab-plane, and (c) space-filling view of corrugated sheets along the b-axis.

3,4,5-TFP X-ray Structure

Components in **3,4,5-TFP** crystallize in monoclinic space group $P2_1/c$ with an asymmetric unit that contains two **3,4,5-TFP** molecules. The grown structure shows assemblies held together by [O-H$\cdots$O] H-bonds (1.89 $\AA$) (Figure S3a). **3,4,5-TFP** molecules form columns in c-plane sustained by face-to-face [$\pi\cdots\pi$] stacking (3.68 $\AA$) (Figure S3b).

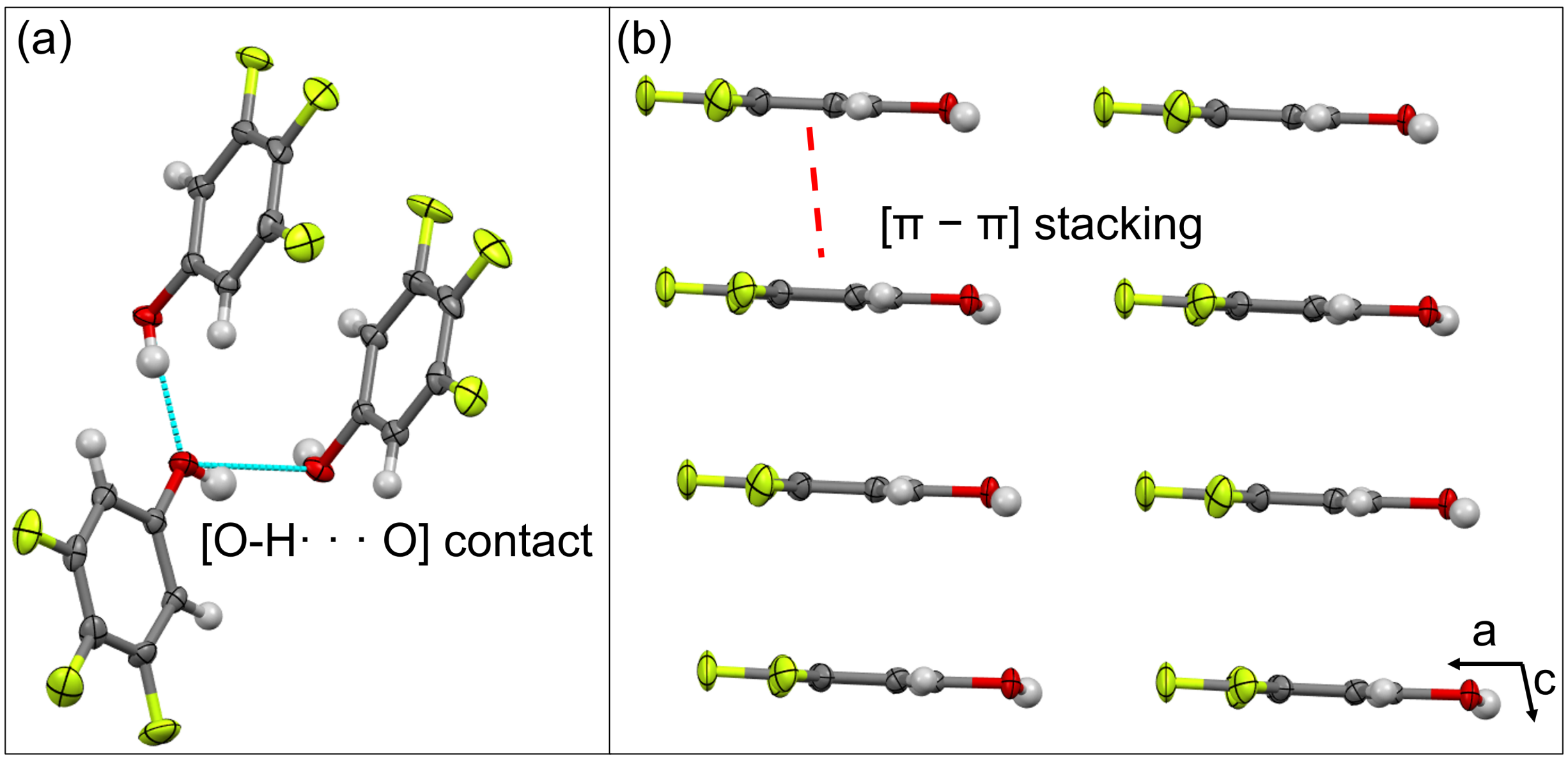

Figure S3: X-ray crystal structure of **3,4,5-TFP**: (a) three-component assembly sustained by [O–H$\cdots$O] hydrogen bonds, (b) columnar aggregation via [π–π] stacking along the c-axis.

S3 Powder X-ray Diffraction Data

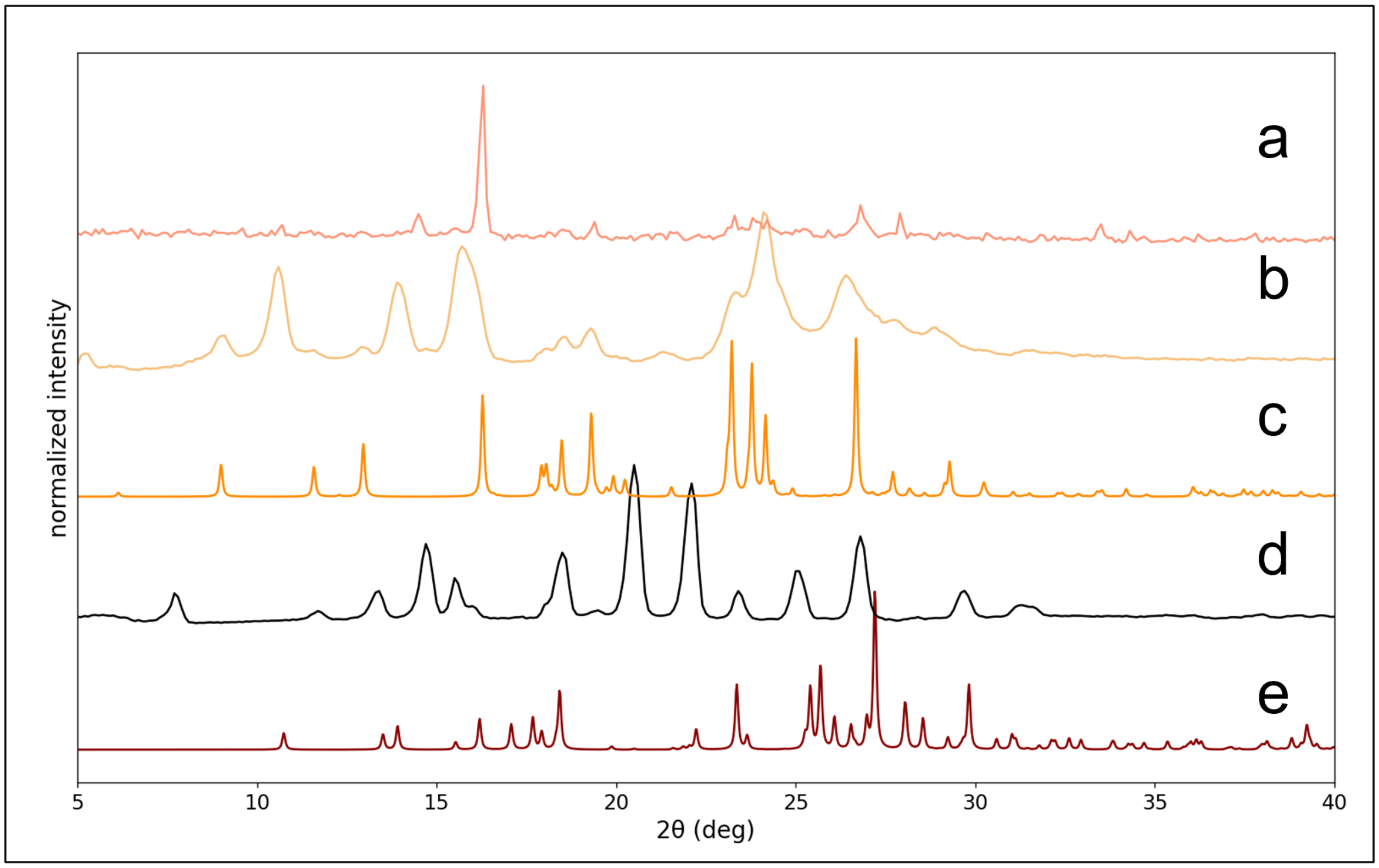

Figure S4: Powder X-ray traces of **BP4VA**·2**(3,4,5-TFP)** co-crystals prepared by (a) solution and (b) sublimation methods, (c) their simulation from single crystal data, and (d) **BP4VA** and (e) **3,4,5-TFP** starting material. The patterns from **BP4VA**·2**(3,4,5-TFP)** obtained via sublimation and solution evaporation are comparable to the simulated pattern from single-crystal data of **BP4VA**·2**(3,4,5-TFP)**, which confirms the presence of the co-crystal phase in the solid.

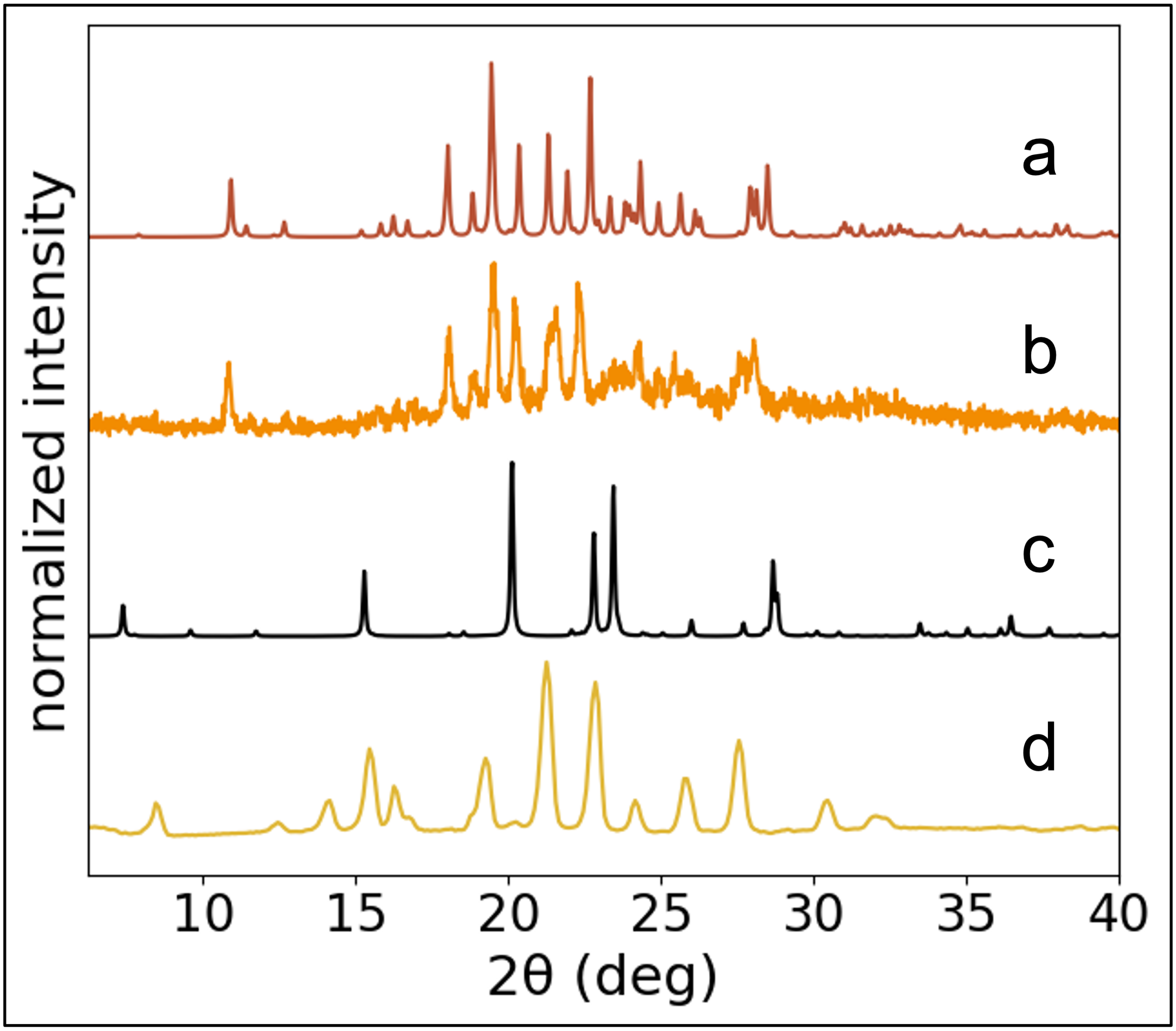

Figure S5: The simulated pattern from (a) single-crystal data of **BP4VA**·2(**PhOH**) is comparable to (b) the pattern from **BP4VA**·2(**PhOH**) obtained *via* **PhOH** sublimation, which confirms the presence of the co-crystal phase in the solid. Starting material traces of (c) **PhOH** (simulated, CCDC: 201623[7]) and (d) **BP4VA** (experimental) are distinct from co-crystal pattern.

S3.1 Scherrer Analysis for BP4VA·2,4,6-TFP

Scherrer analysis was performed on the PXRD pattern obtained after exposing **BP4VA** to **2,4,6-TFP** solid vapor to evaluate peak broadening associated with the surface co-crystal phase. Three peaks assigned to pristine **BP4VA** ($2\theta = 13.40, 20.50, 22.10°$) and three peaks assigned to **BP4VA**·2(**2,4,6-TFP**) ($2\theta = 9.80, 12.22, 16.36°$) were selected for analysis. The FWHM of each peak was determined using Python, and uncertainties are reported as the standard error of the mean. The average FWHM for **BP4VA**-assigned peaks was $(0.34 \pm 0.03)°$, whereas the average FWHM for co-crystal-assigned peaks was $(0.7 \pm 0.1)°$. Thus, the co-crystal-assigned peaks are broader than the **BP4VA**-assigned peaks at approximately the 90% confidence level, consistent with a smaller domain size for the co-crystal phase. Using the Scherrer equation with $K = 0.9$ and $\lambda = 0.15406$ nm, the crystallite size of the co-crystal phase was estimated to be (12 ± 1) nm. This value is reasonably consistent with the surface morphology observed in close-up SEM images of exposed **BP4VA**.

S4 Polarized Optical Microscopy Data

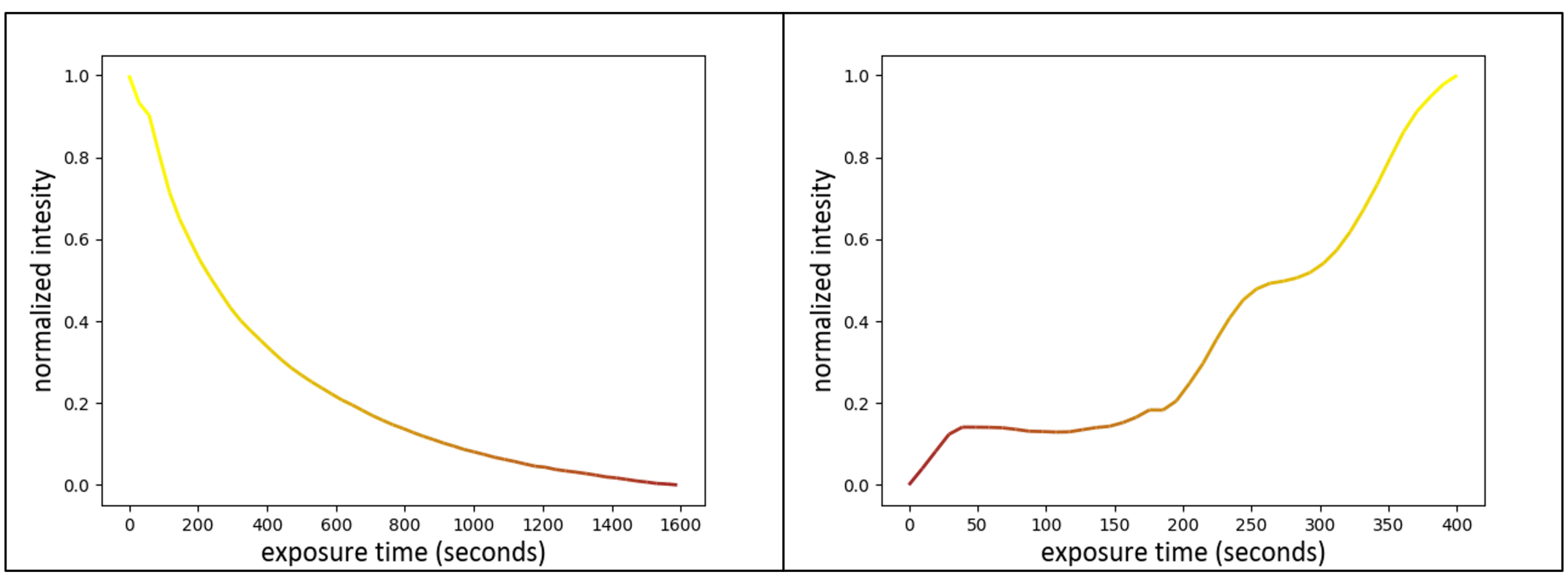

Figure S6: Normalized color intensity vs. time plots of **BP4VA** single crystal after (a) **3,4,5-TFP** exposure, and (b) **BP4VA** thermal recovery. The maximum recovery was achieved at 80°C.

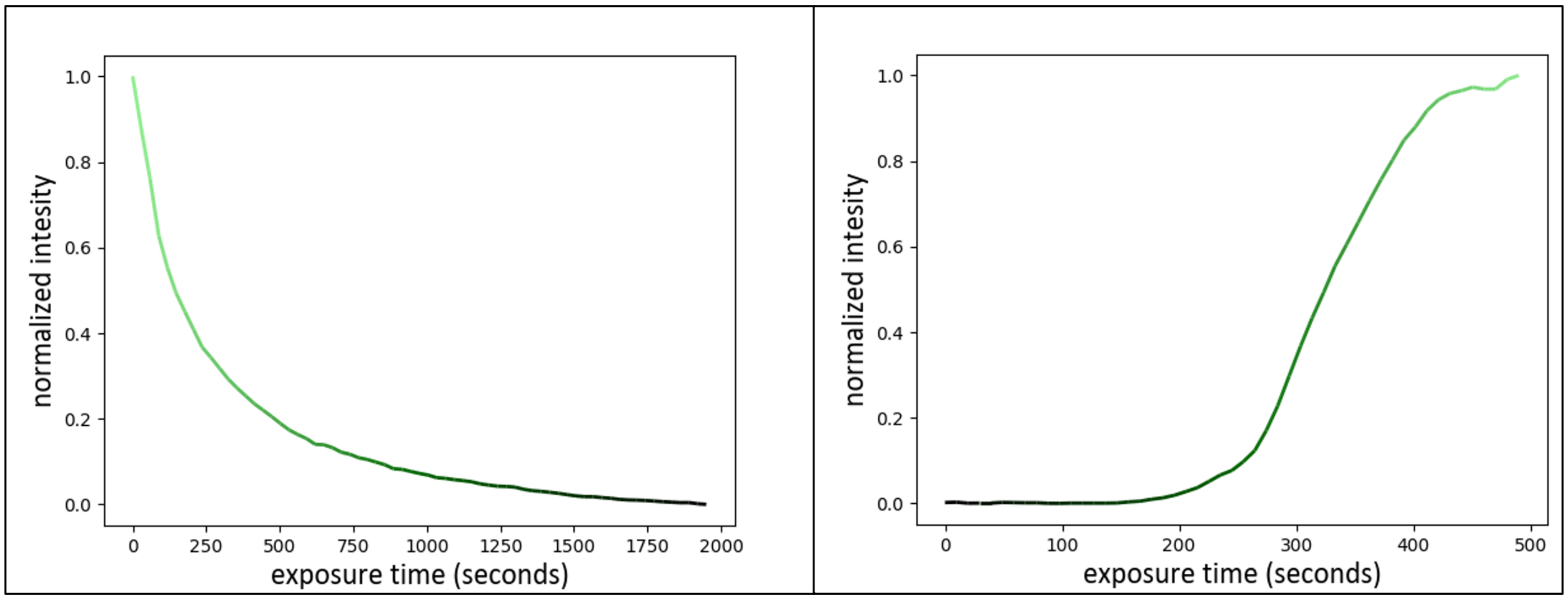

Figure S7: Normalized fluorescence intensity vs. time plots of **BP4VA** single crystal after (a) **3,4,5-TFP**, and (b) **BP4VA** thermal recovery. The maximum recovery was achieved at 100°C.

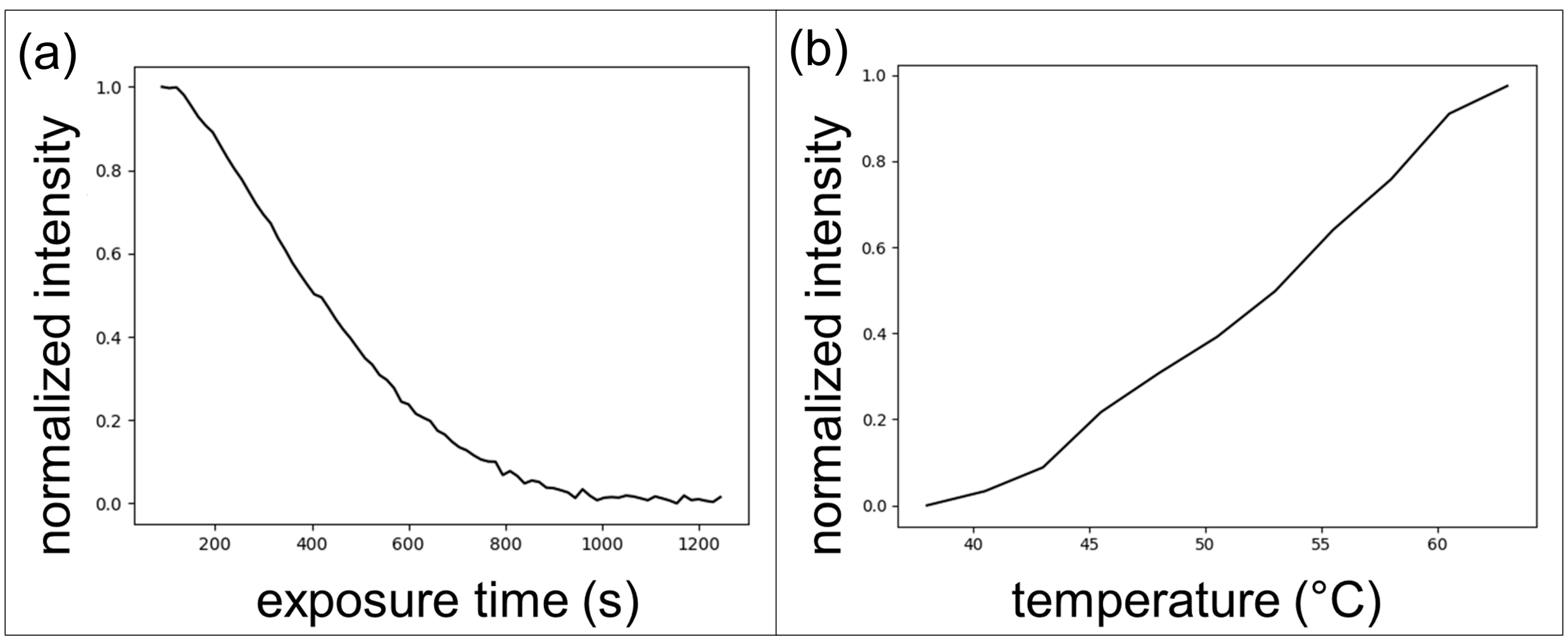

Figure S8: Normalized color intensity vs. time plots of **BP4VA** single crystal after (a) **PhOH** exposure, and (b) **BP4VA** thermal recovery.

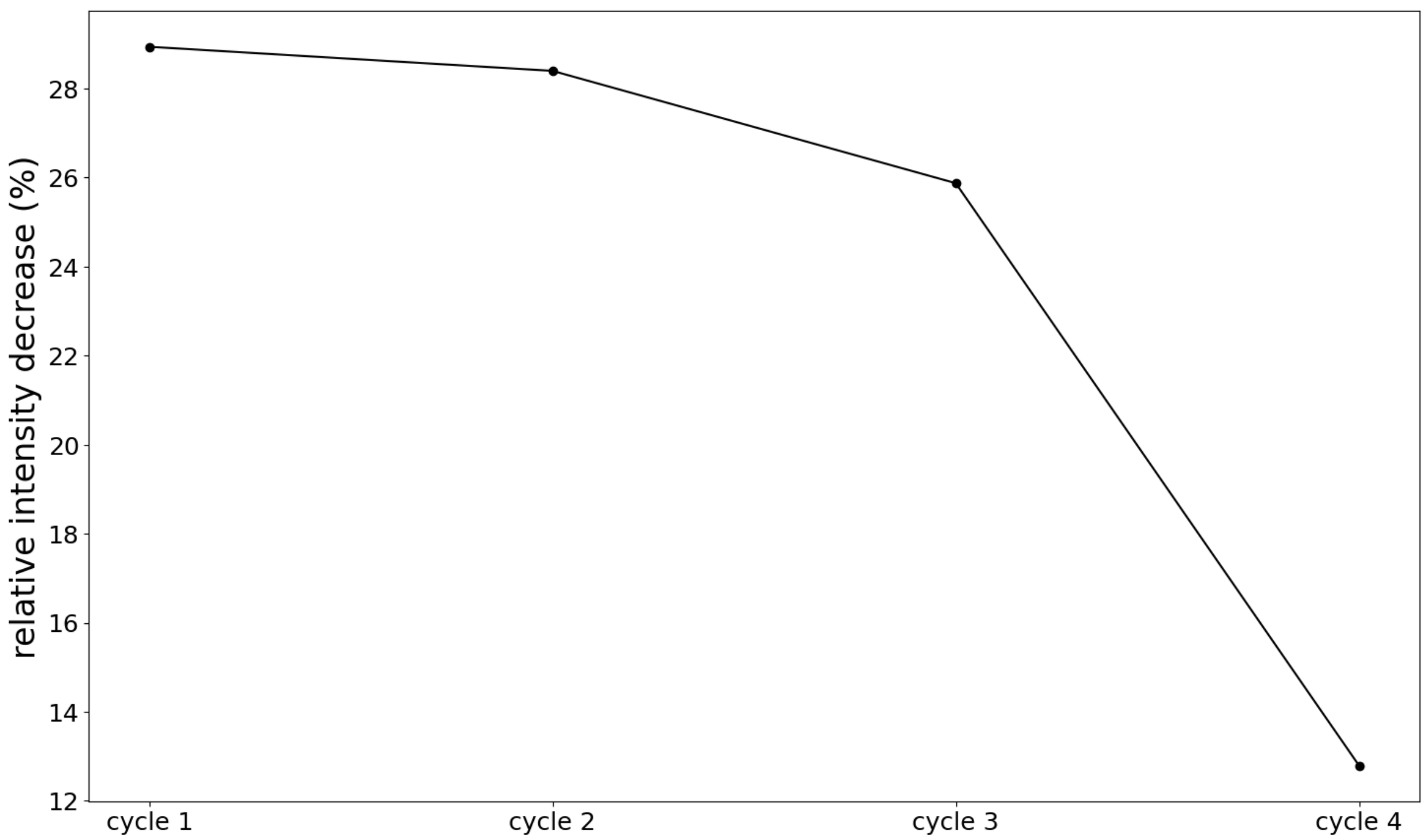

Figure S9: Relative intensity decrease after **2,4,6-TFP** solid vapor exposure vs. the amount of annealing cycles of **BP4VA** single crystal.

S5 Fourier-transform infrared spectroscopy data

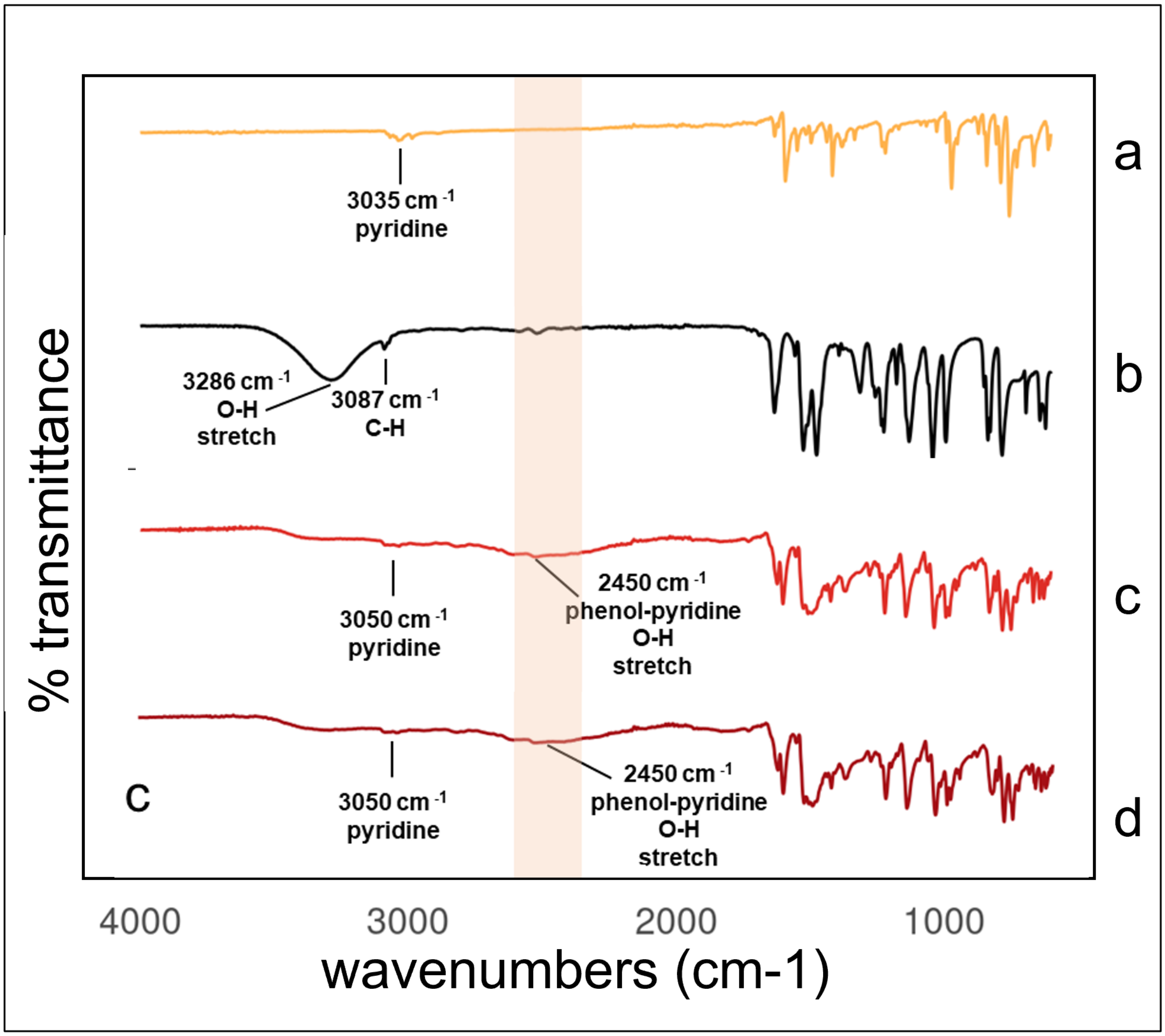

Figure S10: FT-IR spectrum of (a) **BP4VA** and (b) **3,4,5-TFP** starting material and their resulting co-crystal, **BP4VA**·2(**3,4,5-TFP)** prepared by (c) solution and (d) sublimation methods. Relevant peaks for co-crystal formation are highlighted.

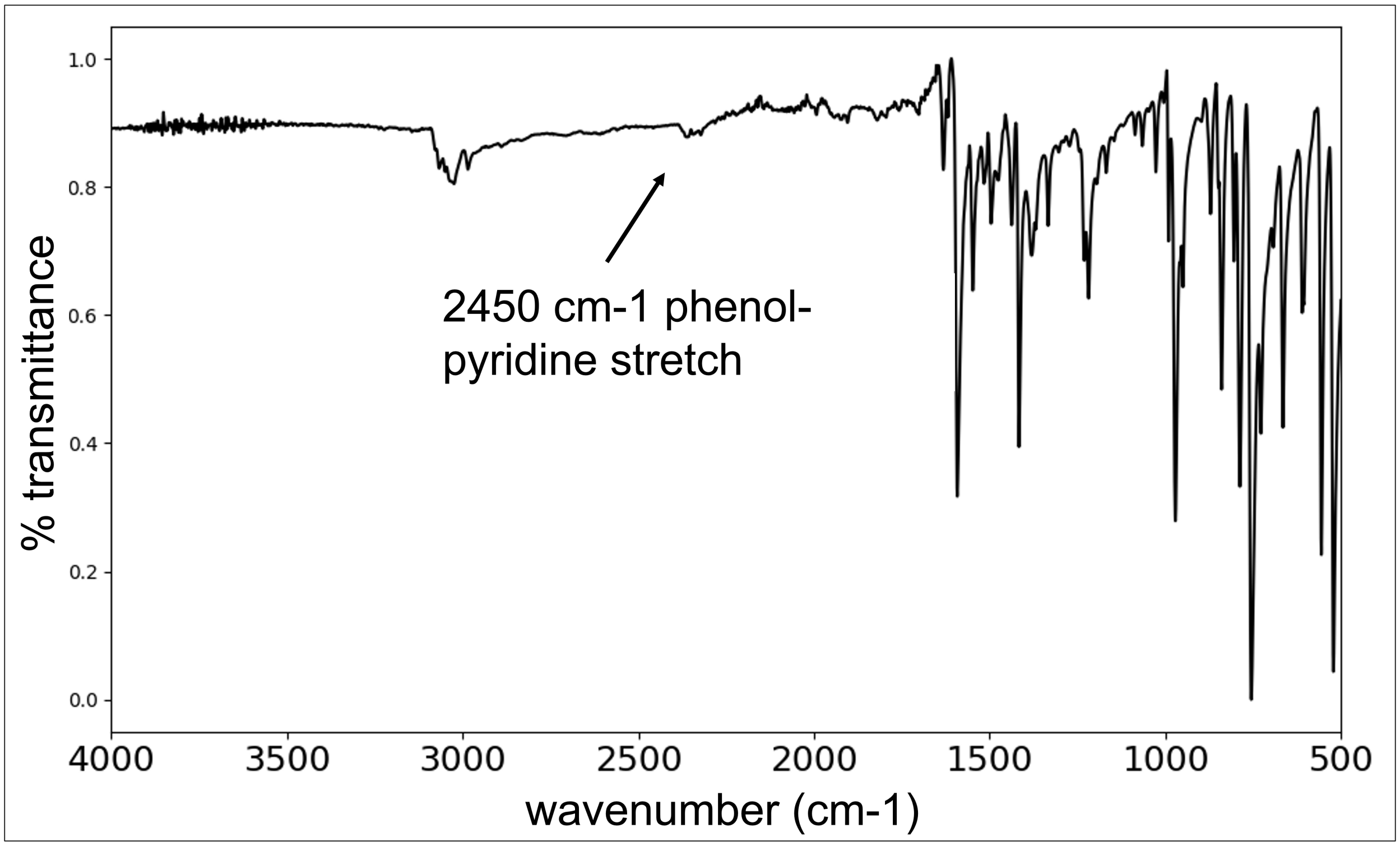

Figure S11: FT-IR spectrum of **BP4VA** exposed to phenol solid vapor.The emergence of the same phenol-pyridine stretch indicates surface co-crystallization. This the similar underlying mechanism for phenol vapor-induced chromic change in **BP4VA** crystals.

S6 ¹H NMR spectral data

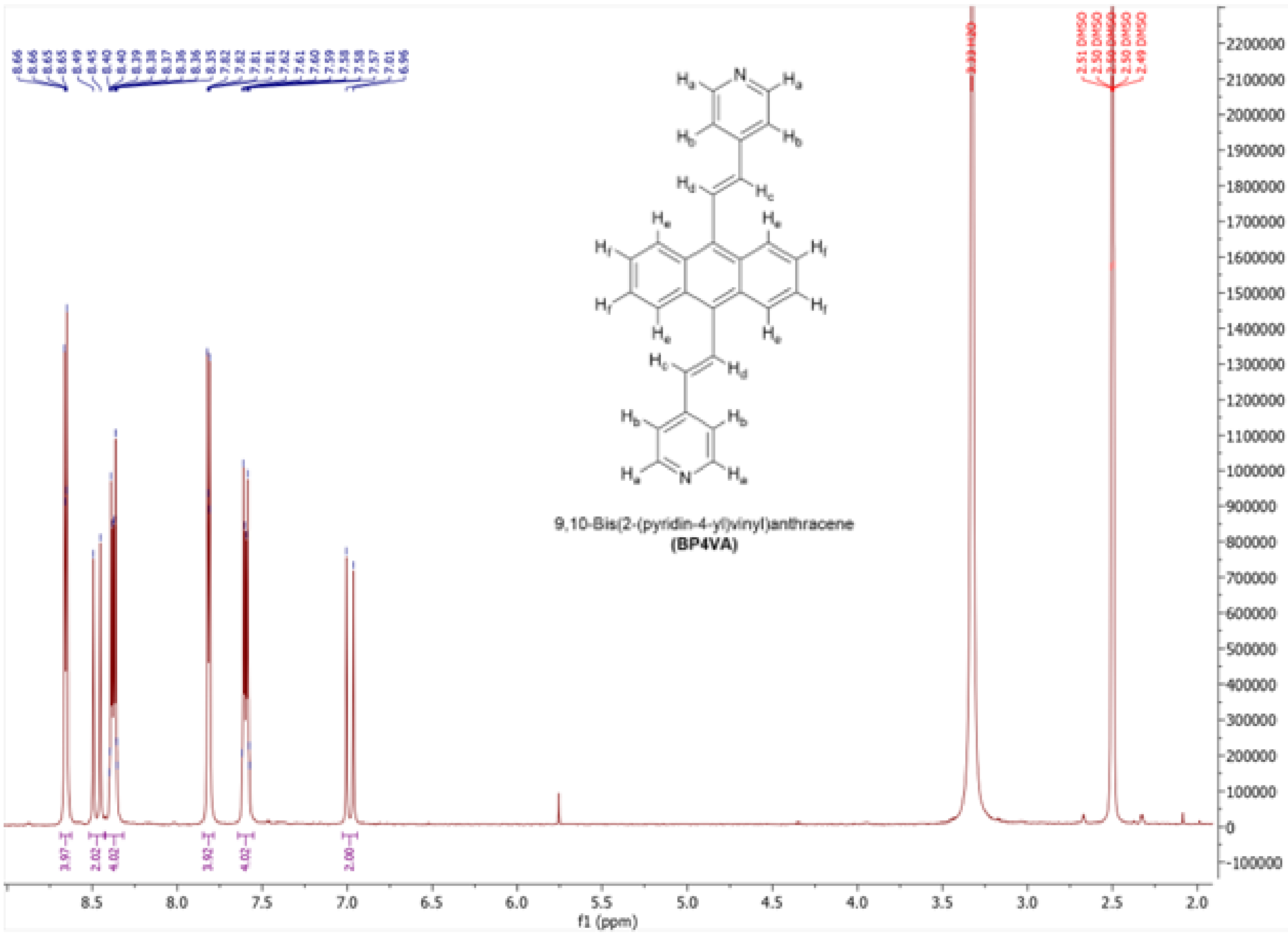

Figure S12: ¹H NMR of **BP4VA**. ¹H NMR (400 MHz, DMSO) δ 8.66 (d, J = 6.1 Hz, 4H, Ar-H_a), 8.47 (d, J = 16.6 Hz, 2H, Ar-H_c), 8.37 (dd, J = 6.0, 3.0 Hz, 4H, Ar-H_b), 7.82 (d, J = 6.0 Hz, 4H, Ar-H_e), 7.59 (dd, J = 7.0, 3.2 Hz, 4H, Ar-H_f), 6.98 (d, J = 16.6 Hz, 2H, Ar-H_d).

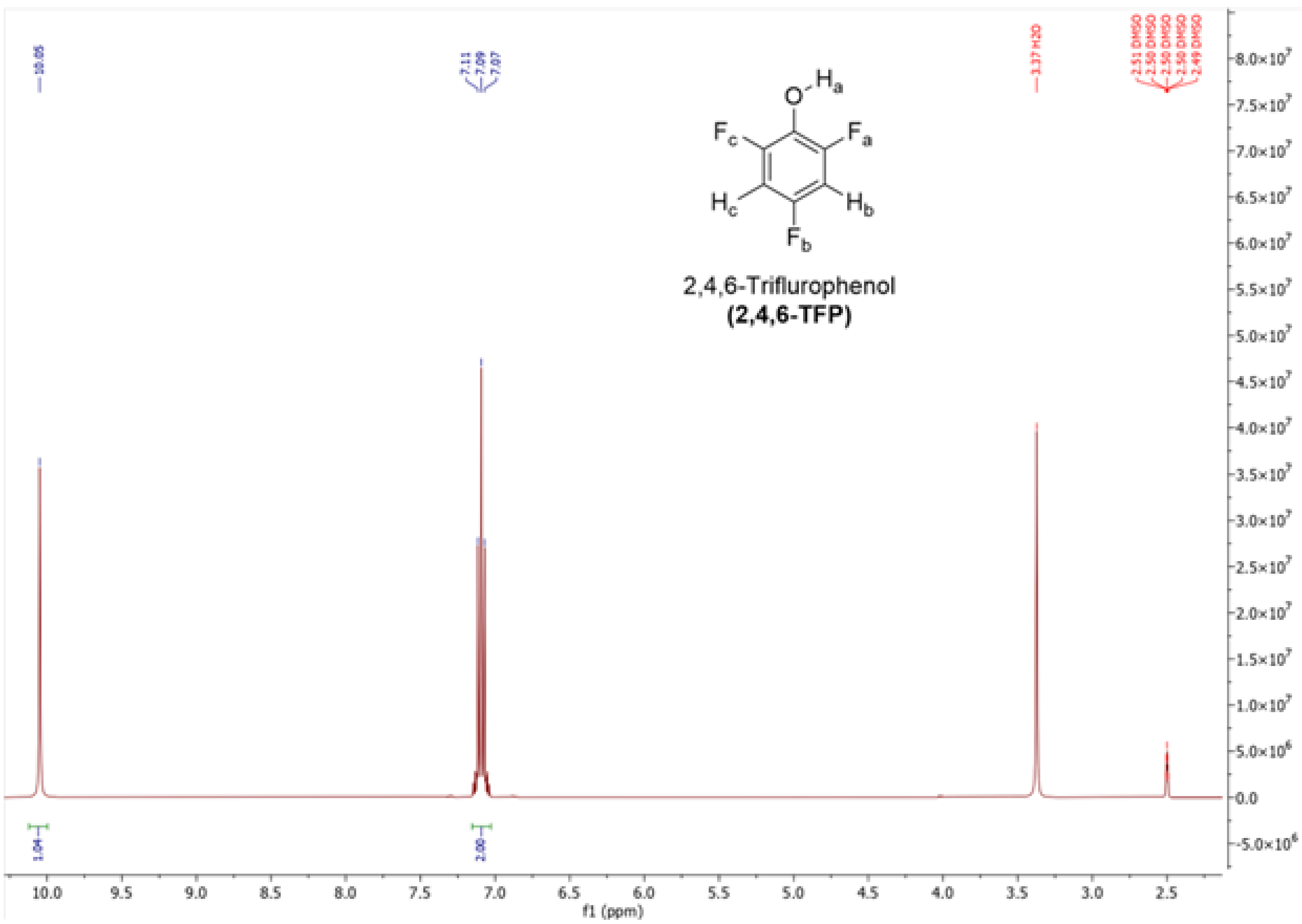

Figure S13: ^{1}H NMR of **2,4,6-TFP**. ^{1}H NMR (400 MHz, DMSO) δ 10.05 (s, 1H, Ar-O-H$_a$), 7.09 (t, J = 8.5 Hz, 2H, Ar-H$_b$).

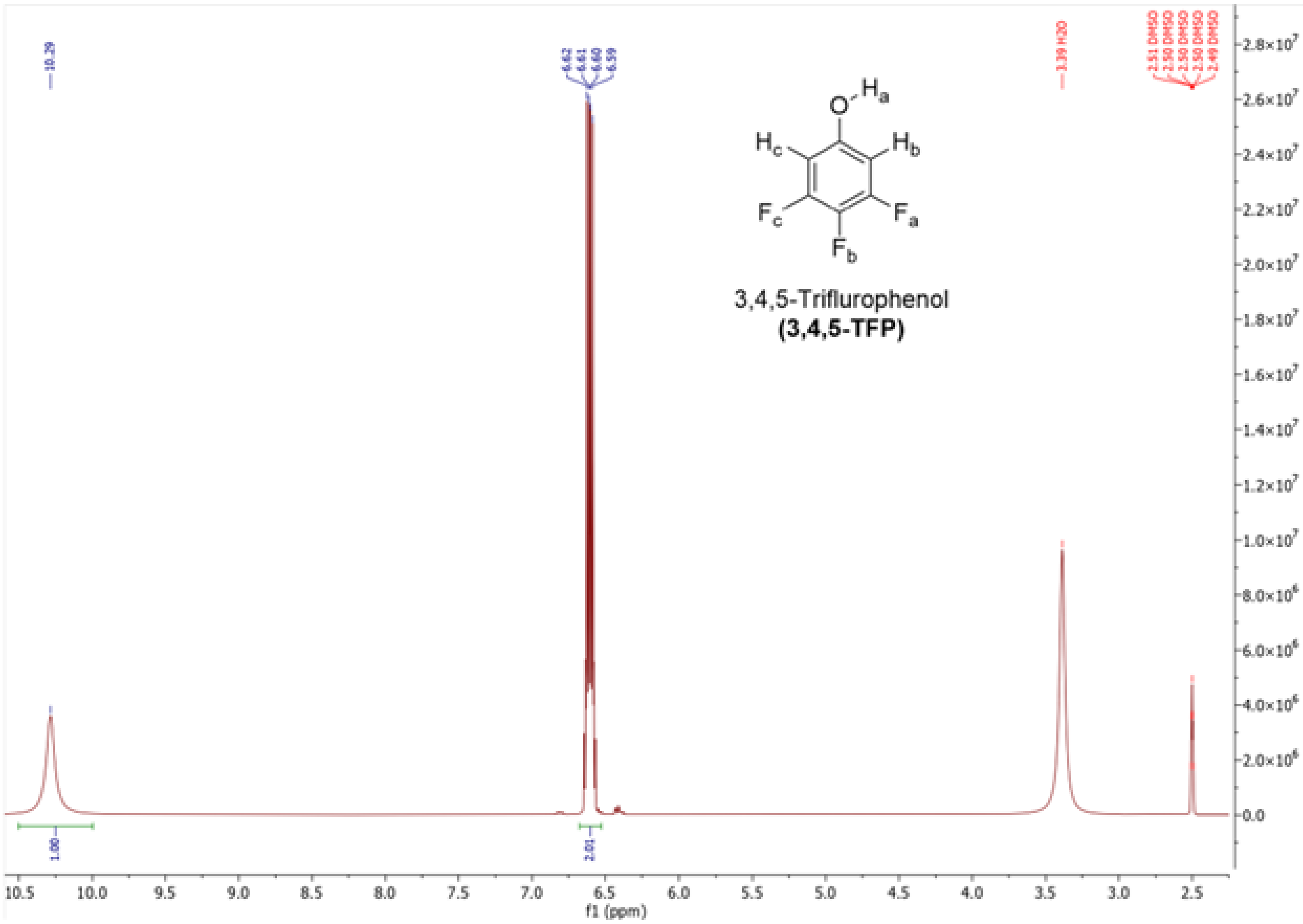

Figure S14: ^{1}H NMR of **3,4,5-TFP**. ^{1}H NMR (400 MHz, DMSO) δ 10.29 (s, 1H, Ar-O-H$_a$), 6.60 (dd, J = 10.2, 6.0 Hz, 2H, Ar-H$_{b,c}$).

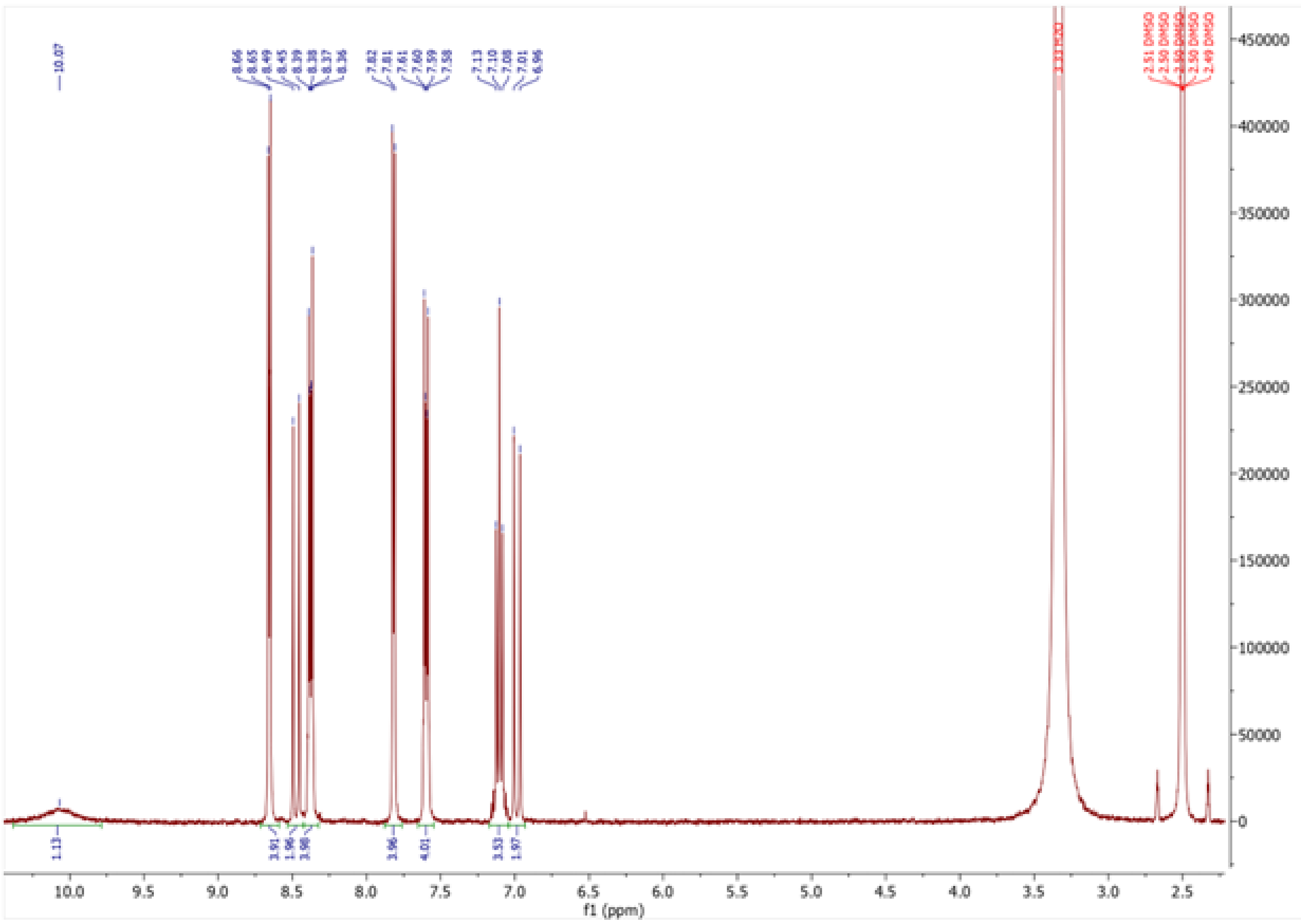

Figure S15: ^{1}H NMR of **BP4VA**·2(**2,4,6-TFP**) obtained by slow evaporation. ^{1}H NMR (400 MHz, DMSO) δ 10.07 (s, 2H), 8.65 (d, J = 5.8 Hz, 4H), 8.47 (d, J = 16.5 Hz, 2H), 8.37 (dd, J = 6.3, 3.2 Hz, 4H), 7.82 (d, J = 6.1 Hz, 4H), 7.60 (dt, J = 6.9, 3.2 Hz, 4H), 7.10 (d, J = 9.8 Hz, 4H), 6.98 (d, J = 16.6 Hz, 2H).

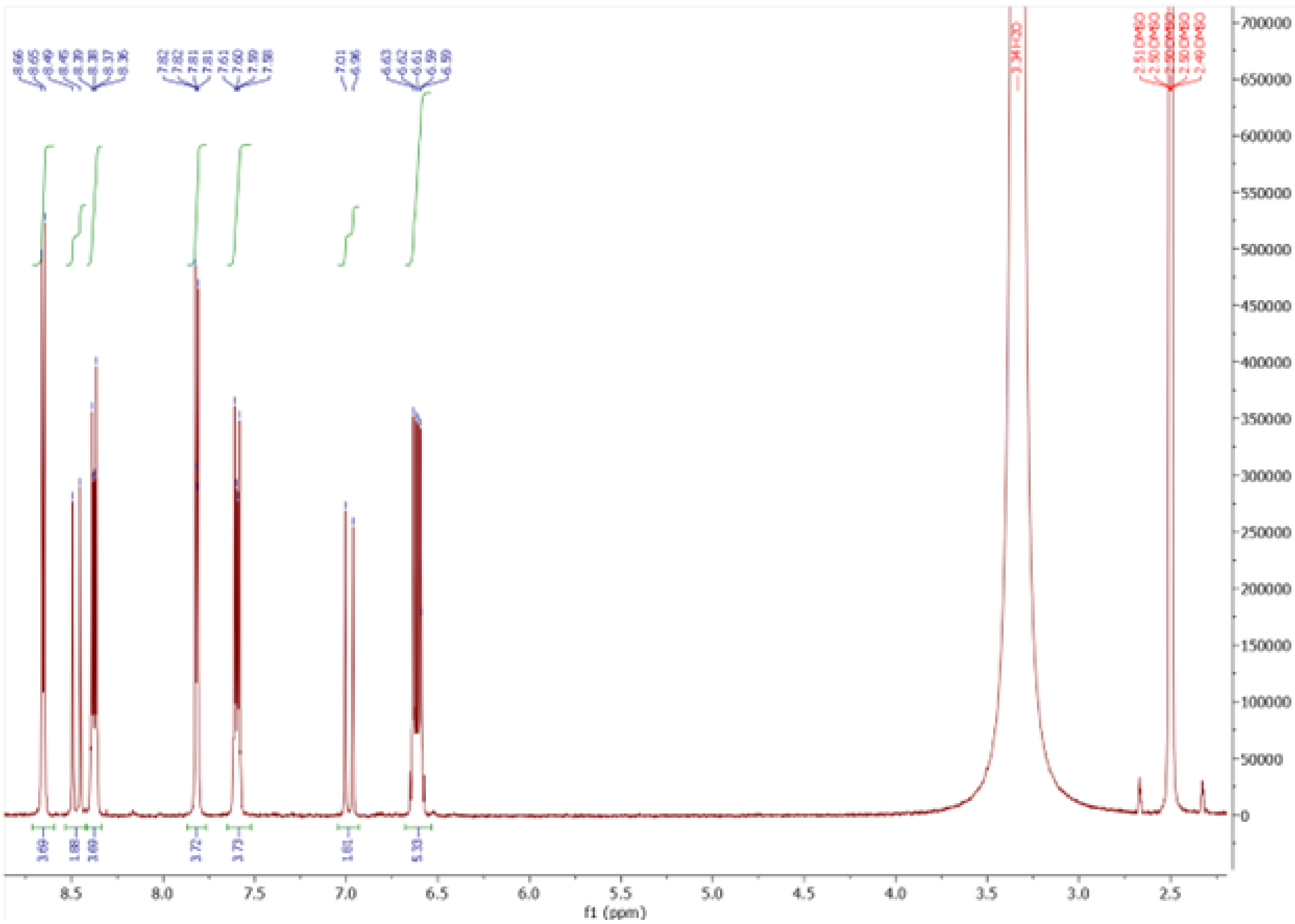

Figure S16: ^{1}H NMR of **BP4VA**·2(**3,4,5-TFP**) obtained by slow evaporation. ^{1}H NMR (400 MHz, DMSO) δ 8.65 (d, J = 6.1 Hz, 4H), 8.47 (d, J = 16.6 Hz, 2H), 8.38 (dd, J = 6.8, 3.4 Hz, 4H), 7.82 (d, J = 5.9 Hz, 4H), 7.59 (dd, J = 6.8, 3.0 Hz, 4H), 6.98 (d, J = 16.6 Hz, 2H), 6.61 (dd, J = 10.5, 5.9 Hz, 6H).

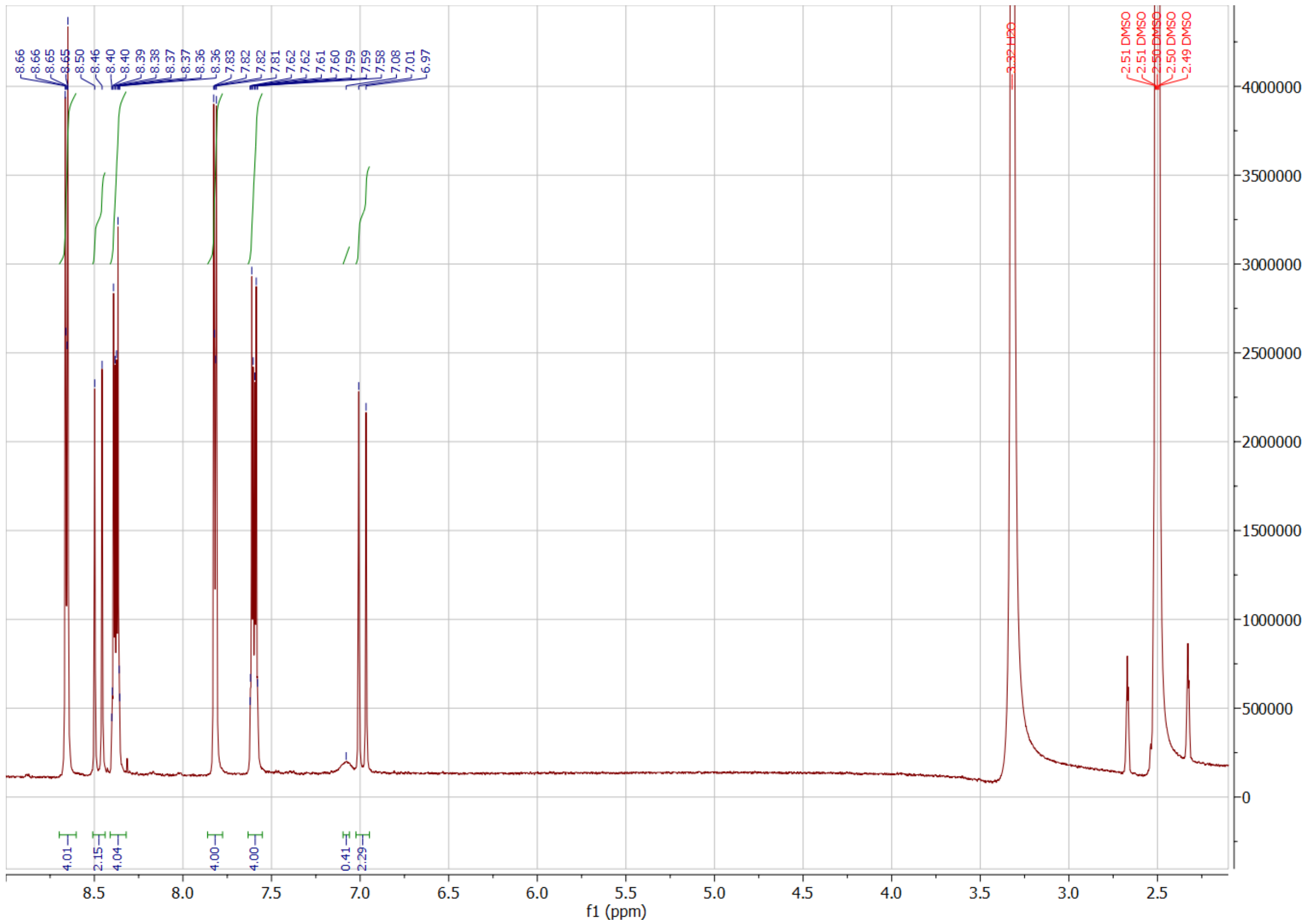

Figure S17: ^{1}H NMR of **BP4VA**·2(**2,4,6-TFP**) obtained by surface co-crystallization. ^{1}H NMR (400 MHz, DMSO) δ 8.65 (d, 4H), 8.48 (d, 2H), 8.37 (dd, 4H), 7.82 (d, 4H), 7.60 (dt, 4H), 7.0 8 (s, 2H), 6.98 (d, 2H). The integration values suggest the approximate stoichiometry of the interaction of **BP4VA** : **2,4,6-TFP** = 20 : 1 (n/n).

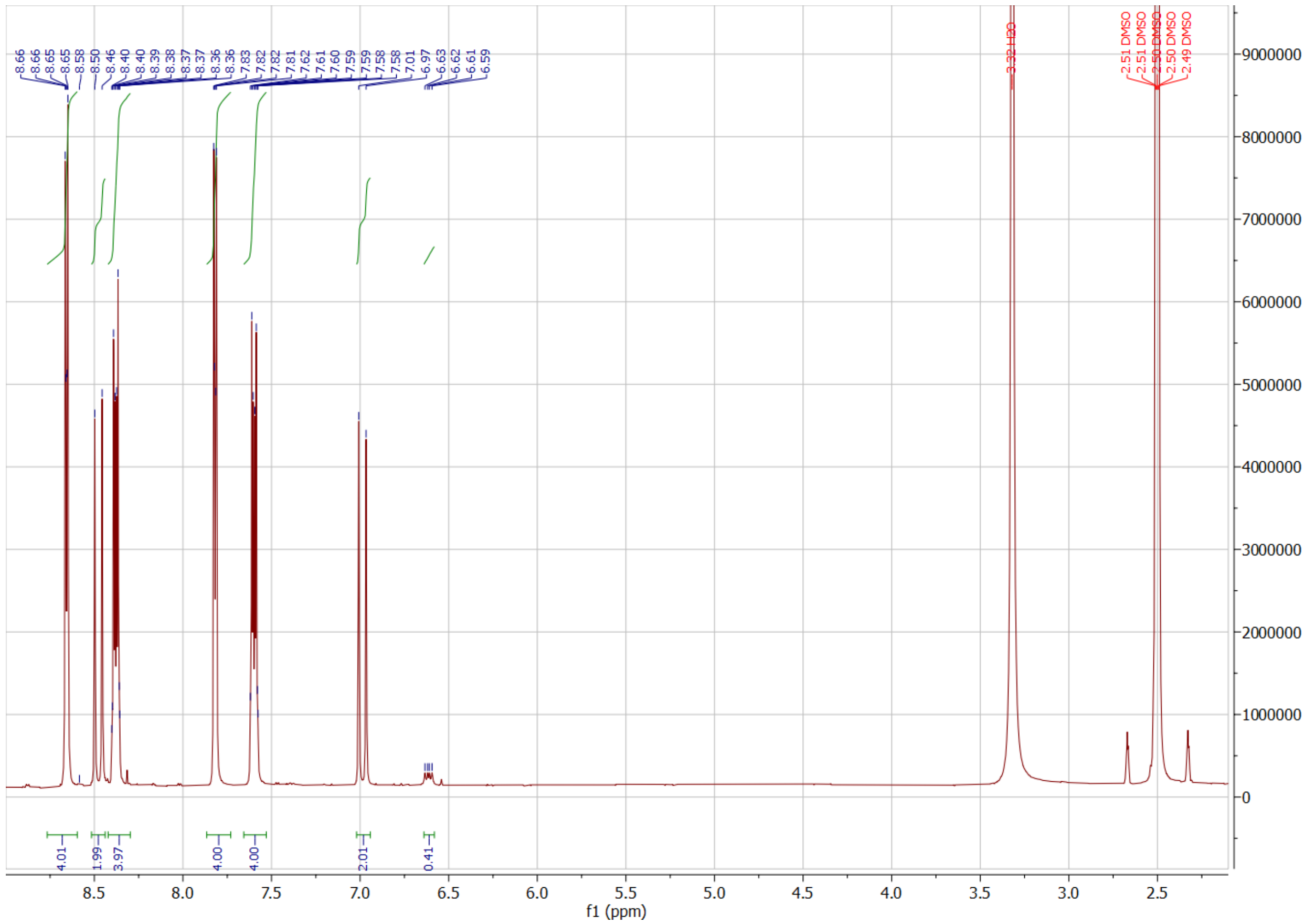

Figure S18: ^{1}H NMR of **BP4VA**·2(**3,4,5-TFP**) obtained by surface co-crystallization. ^{1}H NMR (400 MHz, DMSO) δ 8.65 (d, 4H),8.48 (d, J = 16.6 Hz, 2H), 8.37 (dd, 4H), 7.82 (d, 4H), 7.60 (dt, J = 6.9, 3.2 Hz, 4H), 6.99 (d, J = 16.6 Hz, 2H), 6.62 (dd, 2H). The integration values suggest the approximate stoichiometry of the interaction of **BP4VA** : **3,4,5-TFP** = 20 : 1 (n/n).

S7 SEM Images and EDX Data

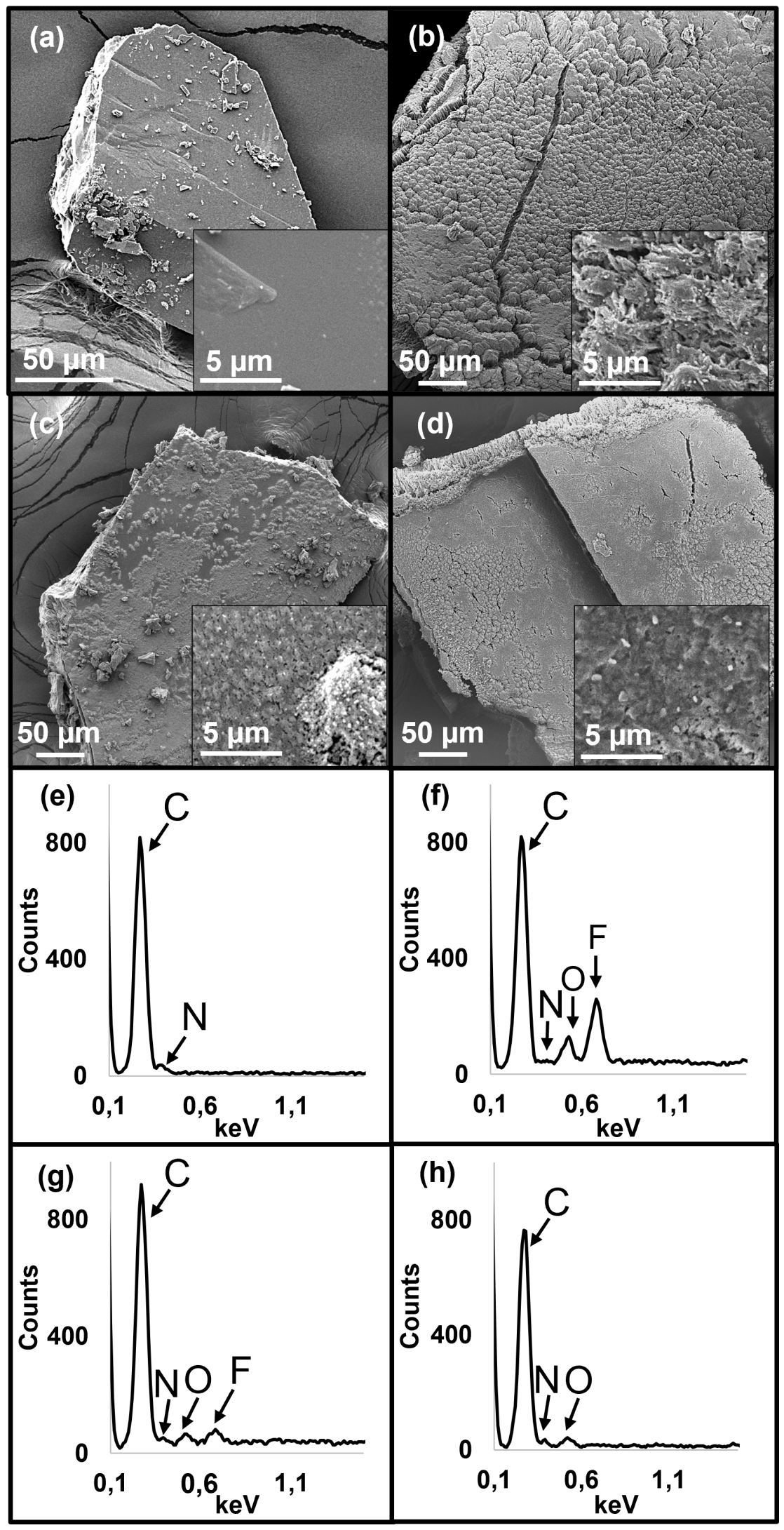

Figure S19: SEM and EDX characterization showing co-crystallization of **BP4VA** with **3,4,5-TFP** and crystals' reversibility. (a) Smooth surface morphology of pristine **BP4VA**, (b) porous and textured surface structure after exposure to **3,4,5-TFP**, (c) reversed surface morphology after annealing, and (d) similarly reversed surface after aging for five months in ambient conditions. (e) EDX spectrum of pristine **BP4VA**, (f) after exposure to **3,4,5-TFP**, (g) after annealing to remove **3,4,5-TFP**, and (h) after aging for five months.

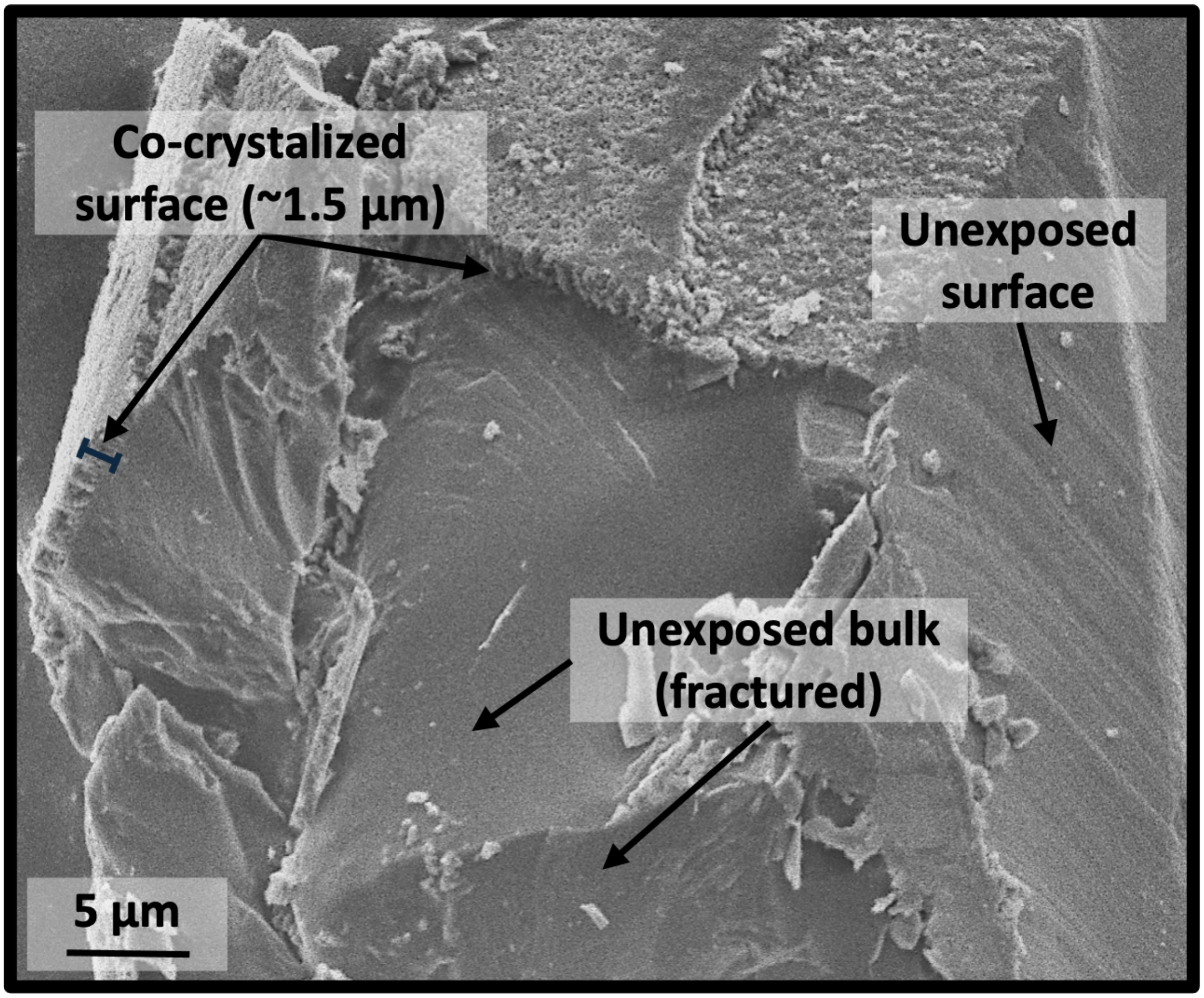

Figure S20: Cross-sectional SEM image of a **BP4VA** crystal exposed to **2,4,6-TFP** vapor. Morphologies consistent with the **BP4VA**·2(**2,4,6-TFP**) co-crystal and pristine **BP4VA** are observed at the exposed surface and in the bulk, respectively. The unexposed surface corresponds to the crystal face that was used for mounting on the glass slide during vapor exposure.

S8 Local Absorbance and Photoluminescence Spectra

Figures S21a and S21b display a brightfield optical micrograph of a light yellow **BP4VA** crystal next to a dark red **BP4VA·2(2,4,6-TFP)** crystal and the corresponding photoluminescence (PL) micrograph collected at $\lambda_{\mathrm{ex}} = 365$ nm, respectively. Both the crystal color and emitted PL appeared redder for the **BP4VA·2(2,4,6-TFP)** crystal compared to the unreacted **BP4VA** crystal. Significant waveguiding was observed for the **BP4VA** crystal, with most emission emerging from the crystal edges. Figure S21c displays local absorption

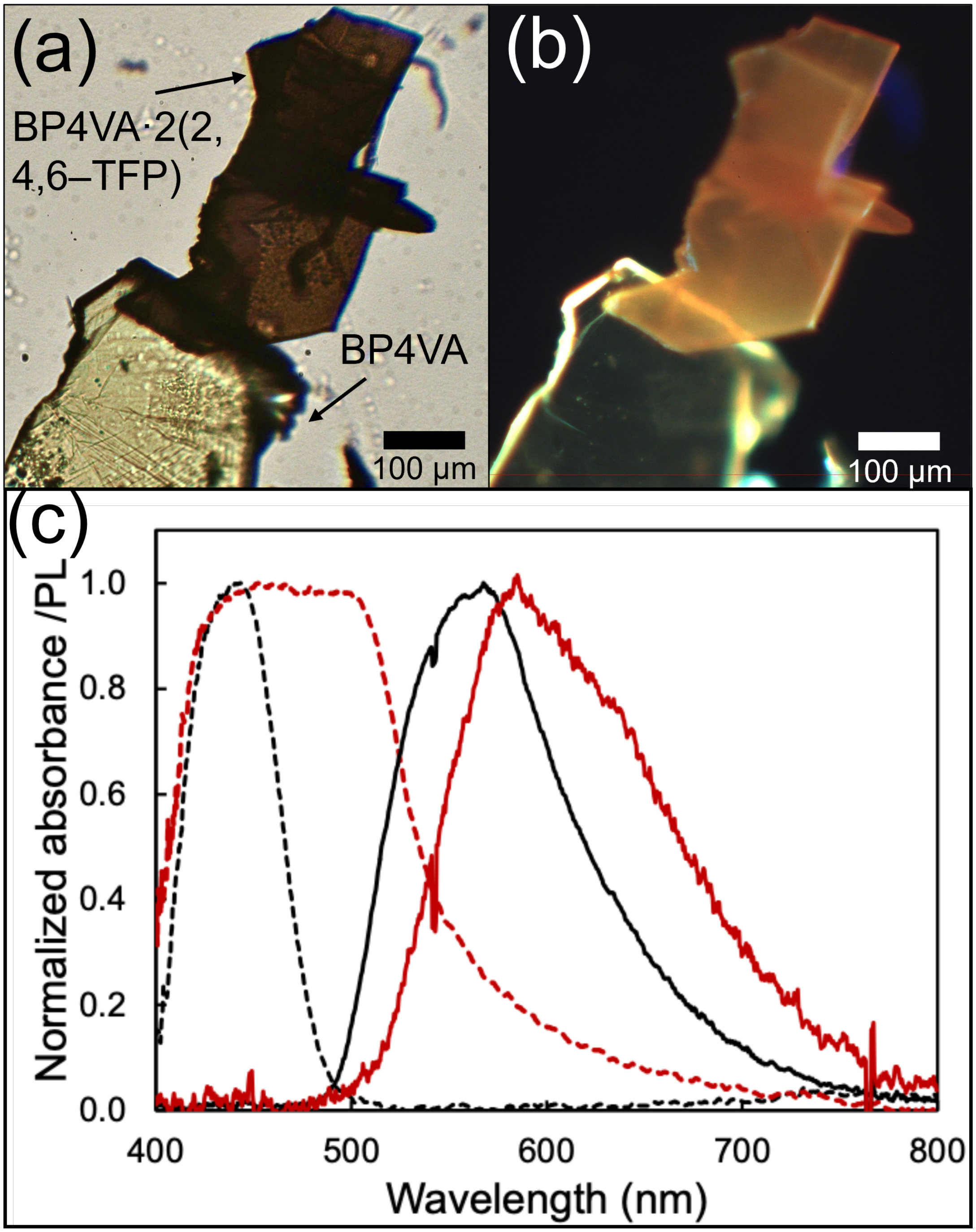

Figure S21: (a) Brightfield and (b) photoluminescence micrograph (λ_{ex} = 365 nm) of a **BP4VA** crystal next to a **BP4VA·2(2,4,6-TFP)** surface co-crystal. (c) Normalized absorbance (dashed lines) and PL (solid lines) of **BP4VA** crystals before (black) and after (red) exposure.

spectra collected on 50×50 μm^2 regions of each crystal. The **BP4VA** crystal exhibited an absorption peak centered at 440 nm, red-shifted by $\sim$ 30 nm from the solution-phase absorption maximum.[1] Upon exposure with **2,4,6-TFP**, the absorption maximum broadened and extended to 500 nm. This shift is indicative of charge transfer (CT) state formation, suggesting co-crystallization as a driver for luminescence decrease.[8–10] Local photoluminescence spectra were likewise collected on 75×75 μm^2 regions of the same crystals and are also shown in Figure S21. The **BP4VA** crystal exhibited an emission maximum centered at $\sim$ 550 nm, in agreement with previous reports.[1,8,11] The emission peak of the **BP4VA·2(2,4,6-TFP)** crystal was red-shifted to $\sim$ 590 nm, also consistent with charge transfer state formation between **BP4VA** and **2,4,6-TFP**.

S9 Thermogravimetric Analysis

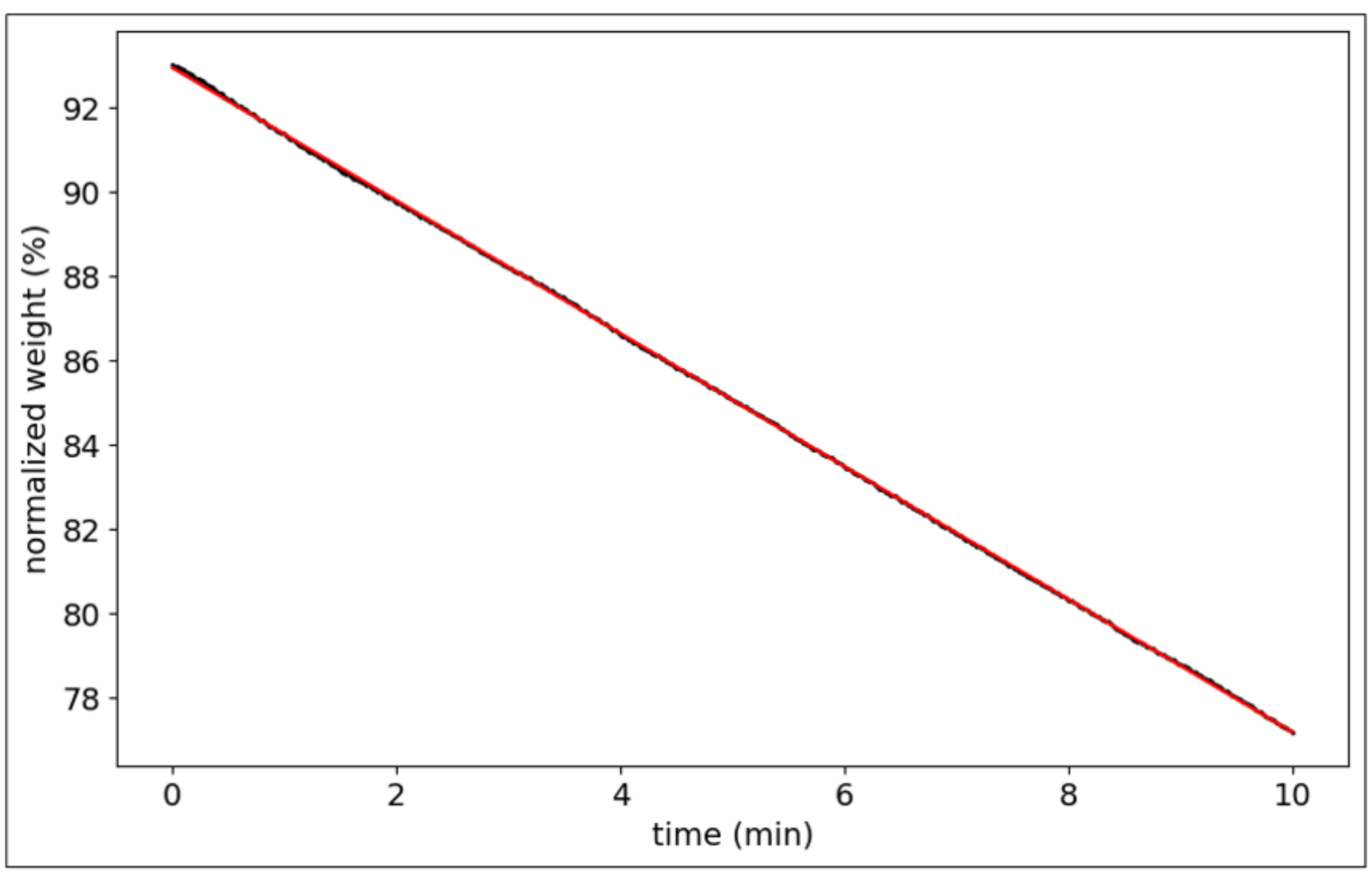

Figure S22: Isothermal TGA reveals the approximate 1.57% $\min^{-1}$ mass-loss (*i.e.,* sublimation) rate for **2,4,6-TFP** under room temperature (295 K). This rate was converted to the amount of material sublimed at the time when the **BP4VA·2(2,4,6-TFP)** surface cocrystal formation can be first visually observed (15 s). Using the experimental chamber dimensions and assuming ideal-gas behavior at 295 K and 1 atm, this calculation gave an estimated detectable **2,4,6-TFP** vapor partial pressure of ≈1 mmHg.

S10 Captions for Movies S1 to S10

Videos were recorded using a Polarized Optical Microscope (POM).

Video S1. POM video showing the sublimation of **2,4,6-TFP** on a single crystal of **BP4VA**.

Video S2. POM video showing the sublimation of **2,4,6-TFP** on a single crystal of **BP4VA**. The POM stage was equipped and irradiated with a UV 365 nm laser.

Video S3. POM video showing thermal annealing of an exposed single crystal of **BP4VA** with **2,4,6-TFP**. The POM stage was equipped with a heating stage.

Video S4. POM video showing thermal annealing of an exposed single crystal of **BP4VA** with **2,4,6-TFP**. The POM stage was equipped with a heating stage and irradiated with a UV 365 nm laser.

Video S5. POM video showing the sublimation of **3,4,5-TFP** on a single crystal of **BP4VA**.

Video S6. POM video showing the sublimation of **3,4,5-TFP** on a single crystal of **BP4VA**. The POM stage was equipped and irradiated with a UV 365 nm laser.

Video S7. POM video showing thermal annealing of an exposed single crystal of **BP4VA** with **3,4,5-TFP**. The POM stage was equipped with a heating stage.

Video S8. POM video showing thermal annealing of an exposed single crystal of **BP4VA** with **3,4,5-TFP**. The POM stage was equipped with a heating stage and irradiated with a UV 365 nm laser.

Video S9. POM video showing the sublimation of phenol on a single crystal of **BP4VA**.

Video S10. POM video showing thermal annealing of an exposed single crystal of **BP4VA** with phenol. The POM stage was equipped with a heating stage.

References

(1) Dong, Y.; Zhang, J.; Tan, X.; Wang, L.; Chen, J.; Li, B.; Ye, L.; Xu, B.; Zou, B.; Tian, W. Multi-stimuli responsive fluorescence switching: the reversible piezochromism and protonation effect of a divinylanthracene derivative. *J. Mater. Chem. C* **2013**, *1*, 7554–7559.

(2) Brennan, S.; Cowan, P. L. A suite of programs for calculating x-ray absorption, reflection, and diffraction performance for a variety of materials at arbitrary wavelengths. *Review of Scientific Instruments* **1992**, *63*, 850–853.

(3) Farrugia, L. J. WinGX suite for small-molecule single-crystal crystallography. *Journal of Applied Crystallography* **1999**, *32*, 837–838.

(4) Sheldrick, G. M. SHELXT—Integrated space-group and crystal-structure determination. *Acta Crystallogr., Sect. A* **2015**, *71*, 3–8.

(5) Sheldrick, G. M. Crystal structure refinement with SHELXL. *Acta Crystallogr., Sect. C* **2015**, *71*, 3–8.

(6) Dolomanov, O. V.; Bourhis, L. J.; Gildea, R. J.; Howard, J. A. K.; Puschmann, H. OLEX2: a complete structure solution, refinement and analysis program. *J. Appl. Crystallogr.* **2009**, *42*, 339–341.

(7) Allan, D. R.; Clark, S. J.; Dawson, A.; McGregor, P. A.; Parsons, S. Pressure-induced polymorphism in phenol. *Acta Crystallographica Section B: Structural Science* **2002**, *58*, 1018–1024.

(8) Campillo-Alvarado, G.; Bernhardt, M.; Davies, D. W.; Soares, J. A. N. T.; Woods, T. J.; Diao, Y. Modulation of π-stacking modes and photophysical properties of an organic semiconductor through isosteric cocrystallization. *J. Chem. Phys.* **2021**, *155*, 071102.

(9) Yoo, S. J.; Kim, J. J. Charge Transport in Electrically Doped Amorphous Organic Semiconductors. *Macromol. Rapid Commun.* **2015**, *36*, 984–1000.

(10) d'Agostino, S.; Grepioni, F.; Braga, D.; Ventura, B. Tipping the Balance with the Aid of Stoichiometry: Room Temperature Phosphorescence versus Fluorescence in Organic Cocrystals. *Cryst. Growth Des.* **2015**, *15*, 2039–2045.

(11) Liu, Y.; Ma, S.; Xu, B.; Tian, W. Construction and function of a highly efficient supramolecular luminescent system. *Faraday Discuss.* **2017**, *196*, 219–229.
